# Effectiveness of Irrigation Protocols in Endodontic Therapy: An Umbrella Review

**DOI:** 10.3390/dj13060273

**Published:** 2025-06-18

**Authors:** Manuel J. Orozco-Gallego, Eliana L. Pineda-Vélez, Wilder J. Rojas-Gutiérrez, Martha L. Rincón-Rodríguez, Andrés A. Agudelo-Suárez

**Affiliations:** 1 Faculty of Dentistry, University of Antioquia, Medellín 050010, Colombia; mjose.orozco@udea.edu.co (M.J.O.-G.); or wilder.rojas@udea.edu.co (W.J.R.-G.); 2 Faculty of Dentistry, CES University, Medellín 050024, Colombia; 3 Faculty of Dentistry, Santo Tomas University, Bucaramanga 681002, Colombia; martha.rincon@ustabuca.edu.co

**Keywords:** root canal irrigants, dental disinfectants, root canal therapy, endodontics, systematic review

## Abstract

**Background:** With the inclusion of evidence-based dentistry, numerous systematic reviews (SRs) and meta-analyses (MAs) have been conducted in endodontics with the best available scientific evidence to improve diagnosis and treatment. **Objective:** To synthesize the scientific evidence on the effectiveness of irrigation protocols in endodontic therapy. **Methods:** Following the umbrella review methodology (UR), a comprehensive literature search was conducted using scientific and grey literature databases. A quality evaluation and a descriptive analysis of the included SRs and MAs were conducted. Quantitative comparability between MAs was carried out. **Results:** Four descriptive SRs and nine MAs were included. Eight articles evidenced high methodological quality. Studies showed the effectiveness and efficacy depending on the study design, the findings of primary clinical trials, and factors related to the type of irrigant, concentration, volume, and irrigation systems. Variability between irrigants and protocols was observed. Follow-up periods extend from hours to years, and there were different study samples. SRs and MAs evidenced limitations regarding methodological aspects. Low overlap of the primary studies was found. Quantitative analyses indicated greater efficacy in microbial reduction and apical healing in favor of passive ultrasonic irrigation (PUI; RD −0.15; 95% CI −0.28, −0.01; *p* = 0.03; I2 = 60%; RD −0.09; 95% CI −0.16, −0.02; *p* = 0.01; I2 = 0%, respectively). **Conclusions:** This UR highlights the importance of root canal disinfection, emphasizing sodium hypochlorite (NaOCl) as the primary irrigant. Enhanced activation methods, such as PUI and lasers, improve irrigant efficiency, while alternatives like chlorhexidine (CHX) offer better biocompatibility. Standardized protocols and evidence-based clinical guidelines are needed. PROSPERO register: CRD42023409044.

## 1. Introduction

For over 40 years, the fundamental role of canal preparation and cleaning in the success of endodontic treatments has been recognized. These processes are interdependent; the diameter of the canals is enlarged using mechanical instruments, facilitating the improved penetration of chemical irrigants to the apical third and complementing the disinfection process [1]. Some studies using high-resolution computed tomography have found that approximately 36% of the total root surface remains unshaped when only mechanical preparation is performed [2]. This is due to the instruments’ limitations in accessing all anatomical variations. Therefore, mechanical treatment should be complemented with irrigating solutions that have an antimicrobial effect and achieve deeper penetration to disinfect the root canal system [3].

Sodium hypochlorite remains the irrigant of choice, primarily because of its unique ability to dissolve organic tissue [4]. Its mechanism of action involves dissociation into hypochlorous acid (HOCl) and hypochlorite ions (OCl-) [5], known as “available free chlorine.” Upon contact with organic matter, this leads to amino acid degradation and hydrolysis, resulting in tissue dissolution. The high pH of the solution (11.5–12.5) further contributes to organic dissolution through fat saponification [6]. It is used during treatment at various concentrations, ranging from 0.5% to 8.5%. However, there is no consensus on the most appropriate concentration for specific diagnoses, as contradictory results regarding its effectiveness have been reported [7,8]. Conversely, as the NaOCl concentration increases, it becomes highly caustic and cytotoxic when it exceeds certain levels and comes into contact with periapical tissues. Several parameters of the irrigant, including the concentration, volume, exposure time, and frequency of exchange during use, may vary in each case, leading to ongoing disagreement on its application in different clinical scenarios [7,9,10].

Chlorhexidine is utilized for disinfection because its cationic molecule binds to negatively charged microbial walls, disrupting the bacterial osmotic balance. At low concentrations, its bacteriostatic activity predominates, while increasing the concentration enhances its bactericidal effect [11,12]. Another characteristic that supports the use of chlorhexidine in endodontics is its substantivity, defined as its ability to prolong antimicrobial activity for several hours. At high concentrations, chlorhexidine exhibits a bactericidal effect likely due to the precipitation and/or coagulation of the bacterial cell cytoplasm caused by protein cross-linking, which results in cell death. At low concentrations, low-molecular-weight substances—specifically potassium and phosphorus—are filtered out, producing a bacteriostatic effect [11,12].

The use of these antimicrobials is complemented by chelating agents, which remove the smear layer generated during instrumentation [13]. This smear layer serves as a potential nutrient source for microorganisms that can interfere with the sealing of dentinal tubules by cements used during obturation [14]. Among the most commonly used chelating agents is the Ethylenediaminetetraacetic acid (EDTA), a colorless, water-soluble acid widely employed to bind di- or trivalent metal ions [15]. It reacts with calcium ions in dentin, forming soluble calcium chelates, and has been found to decalcify dentin to a depth of 20–30 µm [14]. EDTA is used at a concentration of 15–17% and can dissolve hard tissue when applied in the final stages of instrumentation [13]. Although it has little or no antimicrobial effect, it has been shown to have antifungal activity and alter the biofilm matrix, promoting its detachment and enhancing the effect of sodium hypochlorite [16]. However, these agents should not be mixed, as they generate a reaction that rapidly depletes free chlorine, significantly weakening the antibacterial effect [17]. One undesirable effect of using EDTA as an irrigant is its demonstrated reduction in dentin microhardness due to its demineralizing effect, which is why prolonged or excessive use is not recommended [18].

The limitations in the application of irrigants largely depend on the available space and their surface tension [3]. These solutions dissolve both organic and inorganic tissue [19]. They enhance safety during treatment by reducing friction between the instrument and the tooth, lowering the temperature generated by friction, improving the cutting efficiency of the instruments, preventing packing, and avoiding biofilm extrusion into periapical tissues [3]. This facilitates therapy and provides better conditions for safer and more effective treatment.

The development of new techniques aimed at successfully performing effective pulp therapy has led to the emergence of systems designed to enhance irrigation and improve the clinical outcomes of endodontic treatment, such as ultrasonic, sonic, or laser-activated irrigation [20,21,22]. These systems are justified by their ability to improve the action for irrigating solutions in anatomically complex areas, increase the cleaning efficacy, improve the dissolution of organic tissue, eliminate the smear layer, and potentially reduce the microbial load [20,21,22]. However, the available evidence regarding clinical guidelines or standardized protocols for their use remains limited and has largely relied on clinical experience and manufacturers’ instructions.

Currently, with the inclusion of evidence-based dentistry, numerous systematic reviews have been conducted across different clinical specialties to provide clinicians with the best available scientific evidence for improved treatment protocols. From this perspective, when a significant number of systematic reviews and meta-analyses on a specific topic are produced, a useful way to synthesize the evidence is by conducting an umbrella review. This methodology allows for applying the elements and steps of a systematic review through a comprehensive search and descriptive analysis of its results [23]. That is why there is a growing need to expand existing scientific information, since numerous SRs and MAs in the endodontic field have been conducted with inconsistent or inconclusive findings, and an excess of information can confuse clinicians when implementing effective protocols for specific cases.

Accordingly, this study aimed to synthesize the scientific evidence on the effectiveness of the various irrigation protocols used in endodontic therapy.

## 2. Materials and Methods

### 2.1. Design, Study Protocol, and Registration

For study purposes, an umbrella review was conducted following the methodology recommended by the Joanna Briggs Institute (Adelaide, Australia), using a protocol for systematic reviews applied for a comprehensive overview of SR and meta-analysis (SR–MA) research [23]. The protocol for this study was registered at the International Prospective Register of Systematic Reviews (PROSPERO) (Protocol code: CRD42023409044; available from https://www.crd.york.ac.uk/prospero/display_record.php?ID=CRD42023409044; accessed on 16 March 2025) and was approved by the Ethical Institutional Board of the Faculty of Dentistry at the University of Antioquia (Concept N° 160; Act 160/2023). This paper was written in accordance with the PRISMA statement for systematic reviews and meta-analysis [24].

### 2.2. Search Strategy

The following PICO question was used: what is the effectiveness (O) of the different irrigation protocols (I, C) used in endodontic therapy (P)? According to this question, the eligibility criteria for the examined studies were as follows:

▪ Participants: patients in all age ranges, and patients requiring endodontic treatment.
▪ Interventions/control: different types of endodontic irrigation protocols.
▪ Outcome: effectiveness of endodontic therapy (clinical, radiographical, and microbiological).
▪ Study design: we included systematic reviews (SRs) and meta-analyses (MAs). As far as possible, they should accomplish the main criteria established for the Cochrane Collaboration [25] and/or the Centre for Review and Dissemination [26].
▪ Exclusion criteria: other formats such as theoretical reviews, intervention, observational, or analytical studies, critical and theoretical essays, and clinical guides. Similarly, we excluded papers that did not clearly illustrate the irrigation protocol.

### 2.3. Databases and Search Terms

Table S1 (Supplementary Material) shows the main characteristics related to the database sources, search equations, definitions employed for the search strategy according to the MeSH (medical subject headings) terms/thesaurus, and the PICO question.

In summary, we checked four databases containing scientific literature in health sciences (PubMed, Scopus, EMBASE, and LILACS), and we reviewed Google Scholar to search for grey literature. We included papers from all countries and selected those in English, Portuguese, or Spanish through December 2024.

Two reviewers (M.J.O.-G and E.L.P.-V) independently searched for titles and abstracts of potentially eligible articles. If the information met the eligibility criteria, the article was selected for full reading. The reviewers checked the reference list of the articles selected to find further studies not identified in the initial searches. All articles selected for inclusion were processed for data extraction. Disagreements were resolved through discussion and consultation with the other member of the research team (A.A.A.-S, W.J.R.-G, or M.L.R.-R).

### 2.4. Critical Appraisal and Study Analysis

Two of the authors (M.J.O.-G and A.A.A.-S) reviewed the quality of the selected studies. To guarantee the process’ quality, a pilot test with five articles was carried out, and we calculated a simple concordance index with a score of 90%. The AMSTAR-2 tool was used, which is a checklist of 16 items [27]. Each item is answered with yes, partial yes, cannot answer, or is not applicable. Of the possible answers, only yes counts as a point in the total score for assessing the review. AMSTAR-2 characterizes quality at four levels: high, moderate, low, or critically low, according to the guidance document provided by the creators of the instrument.

We carried out a descriptive analysis of the main characteristics of the included reviews: the first author and year of publication, objective(s) of study, journal (name, impact factor according to Scimago Journal Rank, and quartile), number, and type of original articles included in the review, type of study (systematic review or meta-analysis), tool for assessing primary studies, GRADE approach, main results, limitations, and gaps according to the reported findings.

In addition, we checked the overlap of the original studies included for each systematic review (what if means, studies included in several systematic reviews). For that purpose, we used the corrected covered area (*CCA*) index proposed by Pieper et al. [28] and described in methodological terms and implications by Kirvalidze et al. [29]. The mathematical formula for calculating the *CCA* index is as follows:

$$CCA=\frac{N-r}{\left(r\;\times \;c\right)-r}$$

where *N* is the total number of included publications, *r* is the number of unique primary publications, and *c* is the number of systematic reviews.

### 2.5. Comparative Analysis of the Meta-Analyses Included in the Umbrella Review

To provide a comprehensive overview of the clinical, microbiological, and radiographic effectiveness reported in the included studies, a comparative analysis of the meta-analyses (MAs) was conducted. In cases where no overlap was identified between the MA, the original studies were consulted to ensure accurate representation. Conversely, when overlap was present, only the findings from the most recent meta-analysis—or that which included the greatest number of studies—were considered for inclusion. This comparability was assessed across four dimensions: (1) comparison of microbial reduction between passive ultrasonic irrigation (PUI) and conventional irrigation (CI); (2) clinical and radiographic apical healing outcomes between PUI and CI; (3) effectiveness in microbial reduction—defined as the absence of cultivable microorganisms following chemomechanical preparation with chlorhexidine (CHX) and sodium hypochlorite (NaOCl); and (4) endotoxin reduction following chemomechanical preparation with CHX and NaOCl.

For cases with low overlap, Forest plots were used to illustrate individual point estimates with the 95% confidence intervals (95% CI) for each original clinical study, with a diamond symbolizing the pooled point estimate with the 95% CI using the data provided for successful cases of a reduction in microbiological or endotoxin among comparators. Results were pooled using random-effects models and the risk difference (RD), and the mean differences (MDs) with 95% confidence intervals (CIs) were used [25]. The statistical heterogeneity was assessed using the T2, Cochran Q-Test, and I-squared (I2) statistics. Following Cochrane’s recommendations, an I2 statistic below 30% was considered not important, between 30% and 60% was regarded as moderate heterogeneity, between 50% and 90% was considered substantial heterogeneity, and over 75% was considered considerable heterogeneity [25,30]. All analyses were conducted using RevMan (Review Manager, version 5.4.1 software, Cochrane Collaboration, Copenhagen, Denmark).

## 3. Results

**Figure 1:**
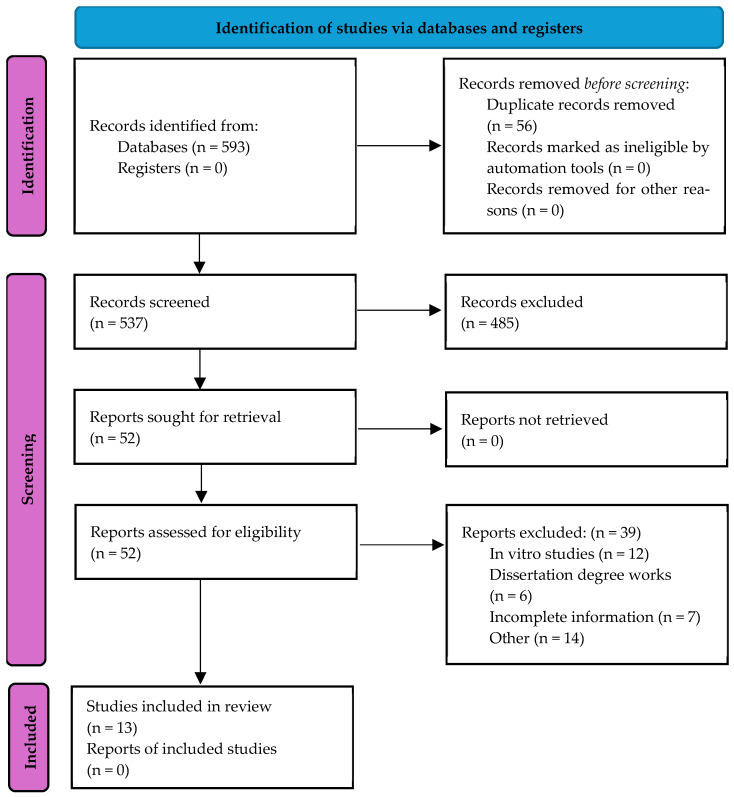
Flow chart of selection process of included studies for the umbrella review. Source: Page MJ et al., 2021 [24].

The initial search resulted in 593 records. After eliminating duplicates, 437 records were selected for revision of the title and abstract, and 52 articles remained for full reading; ultimately, 13 publications were included [31,32,33,34,35,36,37,38,39,40,41,42,43]. Reasons for exclusion are shown in Figure 1.

### 3.1. General Characteristics of the Included Studies

Table 1 shows some general variables related to the included studies. Nine of these studies included an MA [31,32,35,36,38,39,40,41,42]. Six studies were published in journals ranked in Quartile 1 (Q1) according to the Scopus database [32,35,36,37,38,39]. One study was published in a journal not ranked by Scopus [41], and the remaining studies were published in Q2 and Q3 journals [31,33,34,40,42,43]. These SR–MAs included randomized and not randomized clinical trials (RCTs/CTs). The minimum number of original studies included in the SR–MAs was three and a charge of two studies conducted by Silva EJNL et al., 2019 [33] and Gobbo LB et al., 2024 [42]. The maximum number was 17, by the SR carried out by Anagnostaki E et al., 2020 [34]. Four studies did not report if they explicitly received funding sources [33,36,37,41], and one study did not report explicitly if it had conflicts of interest [33]. There exists variability in the countries (including all authors), and it is important to mention that Brazil participated in eight SR–MAs [31,32,33,38,39,40,41,42]. All studies used the Cochrane Collaboration’s tool [25] for the critical appraisal of the RCT/CT [31,32,33,34,35,36,37,38,39,40,41,42,43]. Lastly, eight SR–MAs [33,35,36,38,39,40,41,42] used the GRADE approach (Grading of Recommendations, Assessment, Development, and Evaluation) for assessing the certainty in evidence (also known as quality of evidence or confidence in effect estimates) [44].

### 3.2. Quality Appraisal of the Included SR–MAs

According to Table 1, eight SR–MAs exhibited high quality [35,36,37,38,39,40,41,42], and the remaining five were evaluated as moderate [31,32,33,34,43]. A complete summary of the AMSTAR 2 evaluation of all included studies is presented in Table S2 (Supplementary Material). In the assessment of questions outlined in AMSTAR-2, it was found that item 11 received the highest number of “No” responses (n = 11, 84.6%). Notably, items 1, 3, 5, 7, 8, and 9 emerged with the highest number of “Yes” responses (n = 13, 100%)

### 3.3. Main Findings of SR–MAs According to PICO Question

A summary of the main findings reported in the included SR–MAs considering microbiological, clinical, and radiographic aspects are shown in Table 2. Additional details considering aspects related to the objective of each SR–MA, the various components of the PICO question, the extensive results, and the quality of the evidence according to the limitations of the primary studies are presented in Table S3 (Supplementary Material).

#### 3.3.1. Microbiological Parameters

Microbiological parameters were mentioned in 9 of the 13 included SR–MAs. Two SRs (descriptive) and two MAs showed similar reductions in microorganisms and/or biofilm when conventional irrigation (CI) and passive ultrasonic irrigation (PUI) were used [31,33,35,36]. These four studies evidenced high and moderate methodological quality. Two high-quality studies [37,38] showed better performance in activation methods (PUI). One of the 13 included studies reported a similar reduction in endotoxin levels when chlorhexidine (CHX) gel and sodium hypochlorite (NaOCl) were compared [32], and this study evidenced high methodological quality. One MA was focused on the effect of antimicrobial photodynamic therapy (aPDT) on root canal disinfection (CD). Both protocols (traditional canal root disinfection and combined with aPDT) resulted in a colony-formation unit reduction, but the overall effect was higher after aPDT [40]. Regarding the properties of laser therapy, one study reported antimicrobial efficacy, although the findings reported in this SR are not very specific [34].

#### 3.3.2. Clinical Parameters

Clinical aspects were mentioned in seven of the included SR–MAs, and the findings are divergent. Two SR–MAs reported clinical healing, especially for activation methods [37,42]. One high-quality MA [39] and one moderate-quality SR [43] showed no significant differences when using diode laser irradiation and no adjunct therapy (traditional irrigation). No conclusive results among the irrigation protocols and the pain reduction were reported by two high-quality MAs [35,36]. Finally, a moderate-quality SR was very specific for evaluating the use of a laser in endodontic therapy and showed the efficacy of the irrigation methods in conjunction with laser use, but the results continue to be very descriptive [34].

#### 3.3.3. Radiographical Parameters

Radiographical conditions were mentioned in seven of the included SR–MAs. Similar results in periapical healing were observed in two SR–MAs when comparing both CI and PUI methods [33,35]. However, one high-quality MA [42] showed that the use of PUI resulted in a higher percentage of the periapical healing rate when compared to CI. One SR reported that one primary RCT study that was included showed success in the endodontic treatment when activation methods were used [37]. Two SR–MAs showed no significant differences in healing when diode laser irradiation and traditional irrigation were compared, although there is a tendency to improve periapical healing when laser irradiation is used [39,43]. One moderate-quality SR showed benefits for the use of laser and periapical healing, but the findings were descriptive in manner, and no conclusive evidence remains.

### 3.4. Irrigants and Irrigation Protocols in the Endodontic Therapy

**Figure 2:**
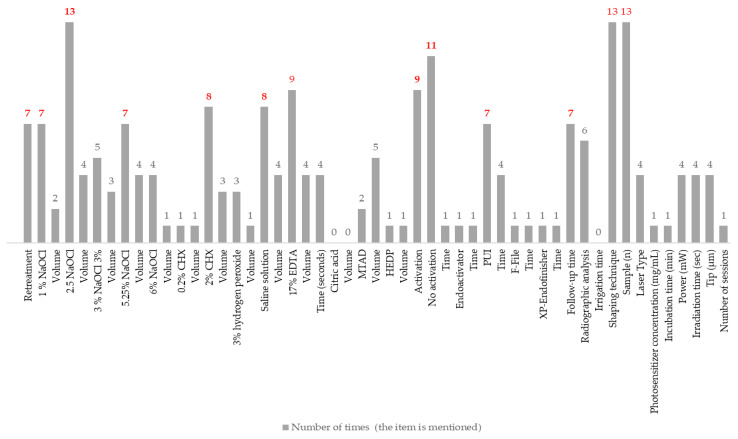
Summary of the irrigants and the protocols used in endodontic therapy according to the reported findings (n = 13). Numbers in red refer to the irrigation protocols and irrigants most frequently mentioned in the included SR-MAs.

Figure 2 summarizes the use of different irrigants and protocols in dental treatments based on findings reported by the included SR–MAs. For detailed information, Table S4 (Supplementary Material) can be consulted. It stands out that NaOCl is the most commonly mentioned irrigant (all SR–MAs included in this umbrella review), especially in concentrations of 2.5% [31,32,33,34,35,36,37,38,39,40,41,42,43] and 5.25% [33,35,36,38,39,40,42]; 2% chlorhexidine (2% CHX) is mentioned in eight studies [31,32,33,35,37,39,41,43]. Saline solution is also mentioned in eight studies [33,35,36,37,38,39,41,43]; 17% Ethylenediaminetetraacetic acid (17% EDTA) is mentioned in nine studies [31,33,34,36,39,40,41,42,43].

Activation methods, such as passive ultrasonic irrigation (PUI), stand out in several studies [31,33,36,37,38,39,42], although some protocols do not mention the activation protocol. The volumes and application times vary significantly across studies, as do the sample sizes. Follow-up periods extend from six months [34] to four years [35], reflecting considerable diversity in the evaluated approaches. Finally, four studies mentioned the laser use with different specifications about the type of device [34,39,40,43].

### 3.5. Scope and Limitations Reported by the Included SR–MAs

Table 3 shows the main limitations of the included studies in this umbrella review. These limitations have been divided based on the methodological considerations in the included primary studies and those related to the quality of the scientific evidence. In the first case, the most reported limitations were that the microbiological assessment considered only the main root canal, mentioned in seven studies [31,33,35,37,38,40,41], and the lack of standardization in techniques, materials, and the concentration of the different irrigants used in endodontic irrigation protocols, also mentioned in seven SR–MAs [32,35,37,38,41,42,43]. In the second case, the substantial heterogeneity of the included primary studies was reported as a limitation in six studies [33,35,36,38,39,41]. Other relevant limitations are observed in this table.

### 3.6. Primary Study Overlap in the Included SR–MAs

For this umbrella review, the 13 SR–MAs included 74 original articles, and the citation matrix is presented in Table S5. Of these, the RCT conducted by Lian YH et al. in 2013 [45] was mentioned in four SR–MAs [33,36,39,42]. The studies conducted by Xavier AC et al. [46], Nakamura VC et al. [47], Morsy DA et al. [48], Tang Z et al. [49], Orozco EIF et al., [50], and Ballal NV et al. [51] were cited three times each, demonstrating the highest level of overlap. Sixteen original studies were cited in two SRs each [52,53,54,55,56,57,58,59,60,61,62,63,64,65,66,67], while the remaining 51 studies were cited only once [21,68,69,70,71,72,73,74,75,76,77,78,79,80,81,82,83,84,85,86,87,88,89,90,91,92,93,94,95,96,97,98,99,100,101,102,103,104,105,106,107,108,109,110,111,112,113,114,115,116,117]. The degree of overlap according to the *CCA* index is 3.3%, and this value indicates “slight overlap”.

### 3.7. Comparative Analysis of Effectiveness Indicators Among Irrigation Protocols Proposed in the Meta-Analyses Included in the Umbrella Review

#### 3.7.1. Comparison of the Microbiological Efficacy Between PUI and CI

**Figure 3:**
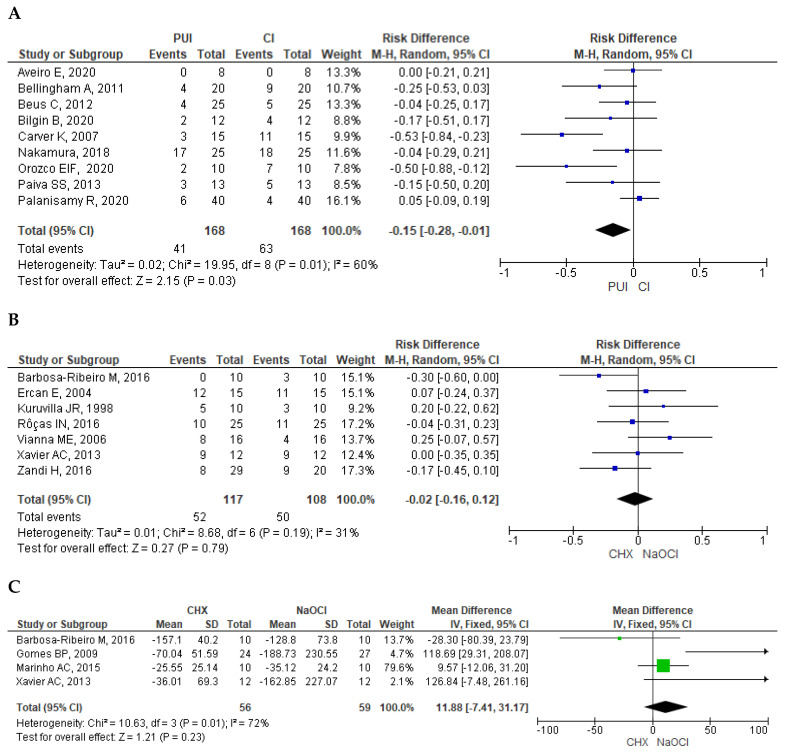
Meta-analysis results. (**A**) Forest plot of microbiological efficacy between PUI and CI. (**B**) Forest plot of the apical healing outcomes between PUI and CI. (**C**) Forest plot of the endotoxin reduction following chemomechanical preparation with CHX and NaOCl. Abbreviations: PUI: passive ultrasonic irrigation; CI: conventional irrigation; CHX: chlorhexidine gel; NaOCl: sodium hypochlorite. P means *p*-value [46,47,50,52,53,54,56,57,58,61,67,70,89,90,96,98,99,113].

To evaluate this aspect, the meta-analyses conducted by Moreira RN et al. [31], Ali NT et al. [36], and Chalub LO et al. [38] were compared. These included nine clinical studies allowing comparability between the two irrigation systems [47,50,52,53,61,70,96,98,99]. The overall result indicated greater efficacy in microbial reduction in favor of the PUI system (RD −0.15; 95% CI −0.28, −0.01; *p* = 0.03; I2 = 60%), although with moderate heterogeneity among the studies (Figure 3A).

#### 3.7.2. Comparison of the Apical Healing Outcomes Between PUI and CI

The MA conducted by Meire MA et al. [39] and Gobbo LB et al. [42]. offers comparability, and they included three original studies [45,49,65], two of them overlapping [45,65]. That is why the MA conducted by Gobbo LB et al. [42] offers more recent findings. General results show that PUI is more successful when compared to CI based on the periapical healing outcome (RD −0.09; 95% CI −0.16, −0.02; *p* = 0.01; I^2^ = 0%). No important heterogeneity between studies was found.

#### 3.7.3. Comparison of the Microbial Efficacy Following Chemomechanical Preparation with CHX and NaOCl

Two MAs offer a comparison for this outcome, Ruksakiet K et al. [35] and Weissheimer T et al. [41], including seven studies [46,57,58,67,89,90,113], two of them overlapping [46,57]. The overall result indicated similar effectiveness between the two irrigants (RD −0.02; 95% CI −0.16, 0.12; *p* = 0.79; I2 = 31%). Moderate heterogeneity among the studies was observed (Figure 3B).

#### 3.7.4. Comparison of Endotoxin Reduction Following Chemomechanical Preparation with CHX and NaOCl

The MA conducted by Neelakantan P et al. [32] and Weissheimer T et al. [41] offered comparability and included four studies [46,54,56,113]. It is important to note that the MA conducted by Neelakantan P et al. included two studies conducted by Marinho AC et al. in 2014 and 2015 [55,56]. The research team decided to include, in the final analysis, the most recent study to verify that both publications have the same study sample [56]. The overall result showed that levels of endotoxin (lipopolysaccharide, LPS) in groups treated with NaOCl were lower than those treated with CHX, but no significant statistical differences were observed (MD 11.88; 95% CI −7.41, 31.17; *p* = 0.23; I2 = 72%). Moderate heterogeneity among the studies was observed (Figure 3C).

## 4. Discussion

This umbrella review demonstrated that, while several irrigation strategies exhibit adequate microbial and clinical efficacy, passive ultrasonic irrigation consistently outperformed conventional methods in terms of apical healing and microbial reduction. However, the lack of standardization across studies and the low overlap among SRs indicate the need for more cohesive research protocols.

### 4.1. Effectiveness, Uses, and Controversies of Irrigating Substances in Endodontic Therapy

The irrigant of choice for endodontic treatment is sodium hypochlorite (NaOCl) due to its antimicrobial activity, organic tissue dissolution capacity, and low cost. The literature indicates that, over the years, it has been used at various concentrations, ranging from 1% to 6%. However, several studies continue to use low concentrations (1–2.5%) due to the risk of accidents during the procedure, where NaOCl might exceed the apical tissues and cause greater damage, as higher concentrations correlate with increased cytotoxicity [118]. Therefore, it is crucial to consider aspects of the procedure that ensure patient safety during irrigation, such as a radiographic analysis, control of needle penetration depth, and the use of appropriate irrigation techniques [118,119,120].

Chlorhexidine (CHX) has been proposed as an alternative to NaOCl primarily for its antimicrobial properties, substantivity, and higher biocompatibility. It is recommended as a final irrigant in endodontic cases with a high bacterial load, where a prolonged antimicrobial effect is required [121,122]. Both CHX and NaOCl exhibit high antimicrobial efficacy. However, despite the similarity in their efficacy, NaOCl remains the irrigant of choice due to its capacity to not only dissolve organic tissue but also facilitate biofilm fragmentation, leading to superior disinfection [123]. In root canal systems with complex anatomies, the efficacy of the antimicrobial activity of CHX may be inferior to that of NaOCl, primarily due to its limited tissue-dissolving capabilities. This limitation may result in overestimating the efficacy of CHX in certain studies, particularly when evaluating the gel formulation. However, these findings should be interpreted with caution, as the primary studies analyzed exhibited several limitations, most notably the inclusion of single-rooted teeth with straight canals. Consequently, the use of CHX gel as a substitute for NaOCl cannot be generally recommended [41].

Regarding the application time and antimicrobial efficacy of the irrigant, clinical studies often do not report this variable, and they do not find significant statistical differences, making consensus difficult and representing a limitation of this review [124]. Nonetheless, in in vitro studies, it has been observed that with hypochlorite, bacterial viability decreases most significantly within 3 min. Beyond this time, its effect diminishes, and substantial microorganism reductions are achieved after 10–30 min of contact with the substrate [10,125]. This underscores the importance of constantly renewing hypochlorite during treatment.

The complexity of the microbial flora within the root canal system is well-known [126]. However, each case can vary widely, making it impossible to determine a single ideal solution for all situations. Thus, adopting an irrigation protocol that achieves maximum disinfection is essential. Although NaOCl has many properties, it may require complementary substances, such as chelating agents, to remove inorganic material. This facilitates smear layer removal and further disrupts the biofilm, which is protected by an extracellular polymeric substance containing Ca+ ions that provide stability, architecture, and resistance. When the chelating agent interacts with the biofilm, it sequesters Ca+ and other cations, disrupting it. This could explain why some studies have found antimicrobial activity for these agents [127,128].

Regarding the application time of chelating agents, clinical studies generally do not assess the effectiveness of this variable in microbiological outcomes. However, in vitro studies have demonstrated that the use of 17% EDTA for varying durations influences the amount of smear layer removed [13]. The volume of EDTA used in the included reviews ranges from 1 to 5 mL. The literature reports that continuous irrigation with 5 mL of EDTA is highly effective for the removal of the smear layer [129] and that increasing this volume does not significantly enhance its effectiveness [128]. It is important to consider that increasing the contact time of EDTA results in significant alterations to the inorganic component of dentin [130]. Reports indicate dentin demineralization to depths of approximately 20–30 µm [131]. Although there is no conclusive clinical evidence to support this [132], the current recommendation is that the contact time of EDTA with dentin—including activation—should not exceed 1 min [133]. Among the studies included in this review, the maximum reported application time was 3 min [41].

The literature reports other substances not involved in regular protocols but observed in isolated studies, such as etidronic acid (HEDP), which acts at a lower pH than EDTA (10.8–12.2) and is a weak alkaline chelator. This characteristic allows for a higher concentration of free chlorine without significantly altering the antibacterial effect of NaOCl when used simultaneously [134]. Additionally, it has been found that the combination of HEDP and NaOCl is more effective for removing *E. faecalis* strains compared to the simultaneous use of NaOCl and EDTA [127]. The combination of HEDP and NaOCl has shown no significant differences compared to the exclusive use of NaOCl [37]. Conversely, MTAD—an irrigant combining a chelator (citric acid) and an antimicrobial agent (doxycycline)—is used for final irrigation. However, it is less efficacious than NaOCl at different concentrations (1–6%) [135]. Additionally, when used in a final irrigation following NaOCl, MTAD demonstrates superior antibacterial effectiveness compared to saline rinses [94].

The heating of sodium hypochlorite has been proposed as part of final irrigation protocols in root canal treatment, due to its potential to enhance the chemical properties of the irrigant [120]. Although this characteristic was not explicitly addressed in the systematic reviews included in this umbrella review, existing evidence suggests that increasing the temperature of NaOCl improves its tissue-dissolving capacity and antimicrobial efficacy [136,137,138,139,140]. This thermal enhancement can be achieved by preheating the syringe or utilizing ultrasonic activation [120]. This topic warrants further investigation and may serve as a relevant focus for future research and systematic reviews specifically aimed at incorporating emerging advancements in the composition, characteristics, and functional properties of endodontic irrigants.

### 4.2. Non-Conventional Irrigating Substances in Endodontic Therapy

Various natural antibacterial agents have been evaluated in the literature as potential alternatives to conventional irrigants. Among them, garlic has demonstrated antimicrobial properties. When combined with lemon, it may yield clinical and radiographic outcomes comparable to those of sodium hypochlorite over follow-up periods of up to 1 year [141]. Neem (Azadirachta indica), a traditional Indian medicinal plant containing more than 140 bioactive compounds, exhibits antibacterial, anti-inflammatory, immunomodulatory, and antifungal properties [142]. In vitro and clinical studies have reported the effectiveness of this plant comparable to that of sodium hypochlorite [143,144,145]. Propolis, which is rich in phenolic compounds, also possesses anti-inflammatory and antibacterial properties; however, studies comparing its efficacy with that of sodium hypochlorite have yielded conflicting results. While some studies suggest similar effectiveness [146], others report inferior outcomes [147].

*Morinda citrifolia* (noni) has demonstrated analgesic, anti-inflammatory, and immunomodulatory properties, along with antimicrobial activity equal to or greater than that of sodium hypochlorite [148,149]. Additionally, its ability to remove the smear layer has been reported [150]. Probiotics such as *Lactobacillus* have recently garnered attention in endodontics due to their anti-inflammatory, antibacterial, and cytoprotective effects. Ex vivo and in vitro studies have shown their high potential to eradicate microorganisms from root canals [151,152,153]; however, the available evidence remains limited. Overall, more than 20 natural products have been investigated as potential endodontic irrigants, some of which have yielded promising results. Nevertheless, the lack of clinical studies with long-term follow-up and standardized methodologies limits their current clinical applicability.

### 4.3. The Effect of Irrigation Systems/Activation Methods in Endodontic Therapy

The effectiveness of irrigants can be enhanced through various activation methods available on the market and evaluated in the literature over recent years. Most studies have found no significant difference in apical periodontitis healing with the use of PUI compared to that of other activation techniques [33,45,47]. This may be because of the many variables influencing healing, making it impossible to isolate activation as the determining factor. Furthermore, microbiological counts have shown no significant differences, likely because molecular techniques detect not only microbial viability but also bacterial remnants, potentially obscuring the activation effects. Consequently, it cannot be definitively stated that PUI provides no improvement.

Primary studies with a low risk of bias and representative samples, as well as meta-analyses, show favorable results for PUI compared to syringe irrigation, supporting its superiority in reducing the bacterial load [36]. This advantage arises from four factors: hydrodynamic phenomena, acoustic microflow and microcavitation, vapor lock elimination (formed by gas bubbles in the apical region that impede irrigant flow), and increased irrigant solution temperature, thus enhancing the efficiency of tissue dissolution and antimicrobials [52,154,155]. Although the available evidence is not conclusive—mainly due to methodological limitations in the reviewed studies—PUI generally demonstrates a statistically significant impact on the healing of apical periodontitis compared to non-activation protocols [42].

Conventional syringe irrigation has some limitations. For instance, it may not fully reach the apical third when using closed-end needles or poses a high risk of extrusion when using open-end needles. In contrast, sonic activation (EndoActivator; Dentsply Tulsa Dental Specialties, Tulsa, OK, USA) removes apical debris without forcing the irrigant into periapical tissues, offering a clinical advantage. However, sonic activation does not significantly reduce the bacterial load compared to conventional syringes [21] and it is considered less effective than ultrasonic activation, as it produces only one node along the length of the instrument, whereas ultrasonics generate multiple nodes that enhance cavitation [37].

Another activation method is the XP-endo Finisher (FKG, La Chaux-de-Fonds, Switzerland), a file system that reacts to temperature changes and adapts three-dimensionally to the anatomy of the canal. This enhances its effectiveness in removing accumulated hard tissue debris, the smear layer, and microorganisms. However, no significant differences in microbial reduction were found when comparing this system to PUI [37]. The F-file (Engineered Endodontics, Menomonee Falls, WI, USA) is another activation method. Coated with a diamond abrasive embedded in a non-toxic polymer tip (20 mm, 0.04 taper), this method removes debris from the dentinal wall and agitates the irrigant. Whereas the F-file was found to be less effective in reducing colony-forming units (CFUs) than PUI or the XP-endo Finisher in the present study, one in vitro study reported higher efficiency with F-file [156], although its clinical efficacy appears to be lower.

Laser technology has gained prominence in dental protocols, especially in endodontics, focusing on disinfection and postoperative pain relief. Lasers with near-infrared wavelengths (810–1064 nm) have photothermal effects on chromophores such as melanin in some microorganisms and penetrate up to 1000 µm into dentin [22,157]. This results in effective microbial destruction without altering adjacent structures. Mid-infrared lasers (2780–2940 nm) create cavitation effects in irrigants, enhancing the removal of microorganisms and biofilms [158]. These lasers have been proposed as an alternative to sodium hypochlorite due to their ability to penetrate dentinal tubules, which is attributable to their high absorption by hydroxyl groups (OH^−^) and water molecules. The cavitation effect generated by mid-infrared lasers using distilled water or a saline solution may substitute for conventional irrigants, such as sodium hypochlorite, yielding comparable radiographic healing outcomes for apical lesions over follow-up periods of up to 12 months [64].

Antimicrobial photodynamic therapy (aPDT) uses a photosensitizer within the canal, irradiated with light of a matching wavelength. This generates reactive oxygen species (ROS) and singlet oxygen (O_2_), causing microbial damage [159].

This umbrella review included a systematic review about laser use, examining postoperative pain, bacterial counts, and apical healing, which found improved outcomes in 14 out of 17 included studies [34]. In this sense, aPDT significantly reduced the periapical index (PAI) at 6 months [78] and demonstrated a positive effect on decreasing the microbial burden after chemo-mechanical disinfection when compared with non-active irrigation, indicating better performance. This suggests that laser therapy may be used as a complementary approach; however, its clinical applicability remains unclear, as numerous variables, such as the type of photosensitizer and light parameters, must be considered. These factors contribute to the current lack of a standardized protocol for the implementation of laser therapy [39].

Another study demonstrated a general positive effect of laser therapy on the healing of apical periodontitis, regardless of the type of laser used. This effect may be because laser therapy can reduce the bacterial load and its biomodulatory action, thus promoting the healing of periapical tissues. Moreover, laser irradiation can effectively eliminate bacteria beyond the apical foramen without the need for extensive apical preparation, as is often required with conventional irrigation methods. Although longer irradiation times enhance the efficacy of laser therapy, the literature generally recommends its application in 20 s cycles, as excessive heat generation may negatively impact periodontal and periapical tissues [43].

### 4.4. Scope and Limitations of This Umbrella Review

It is essential to address certain methodological limitations associated with the primary studies included in this review, particularly those arising from their heterogeneity. The considerable variation in samples, designs, techniques, strategies, and irrigation materials limits the generalizability of some findings and necessitates a cautious interpretation. Nonetheless, this heterogeneity highlights the need and opportunity for further research aimed at the development of evidence-based clinical guidelines. Such guidelines could serve as critical tools to enhance dental practice among professionals in both primary and specialized care settings.

One of the key challenges in conducting an umbrella review is data organization and the need to mitigate the potential overrepresentation of primary studies that are frequently cited or duplicated across systematic reviews. In this study, the corrected covered area (CCA) index was below 5%, indicating a low degree of overlap. However, as highlighted in the relevant literature, this metric should be interpreted with caution. It is crucial to consider the specific features of each systematic review, the main findings reported by the authors, and the overall level of evidence to support robust and informed clinical decision-making.

It is important to comment on the comparability, in quantitative terms, carried out with the MA of this umbrella review. While the research team consulted the information reported by the primary clinical studies, the comparison and final forest plots were carried out with comparable data reported by the MA authors, and even though it is assumed that the reported information is reliable, the possibility of an information section that could underestimate the results is not ruled out. This implies that the results should be interpreted with caution. However, by analyzing the descriptive and qualitative information found in the RS–MAs selected in this umbrella review, it is possible to deduce that the information related to the protocols fulfills the objectives of this investigation.

Another limitation identified among the included primary studies across the various systematic reviews was the methodology used to assess the antimicrobial effectiveness. In some studies, samples were collected using needle and syringe aspiration [94], while in others, they were obtained through absorption with paper points [21,51], followed by bacterial isolation, cultivation, and identification. These methodological differences may compromise the accurate representation of the microbiological content within the root canal system, potentially leading to the loss of critical information regarding the true antimicrobial effect.

It is important to mention that the new shaping protocols that include passive ultrasonic activation (PUI) and laser activation have shown, in most clinical studies, high effectiveness in reducing the bacterial load, as evidenced by lower culture counts and improved clinical symptoms. However, it is particularly challenging to assess bacterial reduction by root canal thirds in clinical settings—an aspect that can be more accurately evaluated in vitro studies, which fall outside the scope of this review.

### 4.5. Clinical Implications and Future Directions for Research

Most of the studies consider only cultivable microorganisms, which limits the detection of other species present in the endodontic microbiome. However, other investigations have employed molecular techniques, such as polymerase chain reaction (PCR), which allow for the detection of a broader range of microorganisms [57,58]. Nonetheless, this methodology has the drawback of identifying DNA from non-viable microorganisms, potentially masking the actual effects of irrigants. Therefore, further studies employing advanced molecular microbial diagnostic techniques are needed to enhance the accuracy of antimicrobial effectiveness assessments.

Regarding the clinical follow-up duration, the included studies reported a mean follow-up time of 6–12 months, with one study extending up to 4 years [35]. In assessing clinical efficacy, variables such as the quality of obturation, coronal seal integrity, and anatomical complexity were not consistently considered. Furthermore, many studies failed to report the type of canal-shaping technique used, although they mostly employed either rotary or manual instrumentation. Additionally, there is insufficient information regarding the apical preparation size, working length determination method, irrigation needle gauge, and depth of needle penetration. Irrigation protocols should vary according to the clinical case diagnosis; however, not all reviews explicitly address key aspects such as the volume of irrigant used, the depth of irrigation needle penetration, and the shaping system based on the complexity of the internal dental anatomy. These gaps highlight the absence of a standardized shaping protocol across studies.

The cleaning and disinfection process is further complemented by using various intracanal medicaments, selected based on the microbial flora present within the root canal system and the periapical diagnosis. These medicaments may play a significant role in enhancing the antimicrobial effectiveness; therefore, dedicated systematic reviews and intervention studies are necessary to determine their true impact.

An area of growing relevance in evidence-based dental practice concerns the development and implementation of clinical guidelines grounded in the best available scientific evidence and their applicability in routine dental care. In the field of endodontics—particularly concerning irrigation systems, protocols, and irrigating solutions—such guidelines represent a critical instrument for standardizing procedures, enhancing clinical outcomes, and guiding informed therapeutic decisions for both general practitioners and specialists within public and private dental care settings. The formulation of these guidelines, therefore, necessitates rigorous research involving expert consensus, a continuous evaluation of emerging evidence, and a sustained commitment to promoting research about safe, effective, and patient-centered clinical practice.

## 5. Conclusions

The findings of this umbrella review underscore the critical role of sodium hypochlorite as the primary irrigant in endodontic therapy, with enhanced outcomes when activation methods such as passive ultrasonic irrigation are used. While chlorhexidine remains a viable biocompatible alternative, its efficacy is comparable but not superior to NaOCl. The field would benefit from standardized clinical protocols and more robust comparative trials to refine best-practice guidelines.

## Figures and Tables

**Table 1:** Main characteristics of the included SR–MAs (n = 13).

First Author, Year	Journal	IF (2023)	Quartile (2023)	Country (All Authors)	MA	Type of Included Studies	Number of Included Studies	Funding Reported	Conflict of Interest Statement	Tool for Assessing Primary Studies	GRADE Approach	AMSTAR- 2
Moreira RN, 2019 [31]	Acta Odontologica Scandinavica	0.569	Q2	Brazil	Yes	RCT	5 (SR) 3 (MA)	Yes	Yes	Cochrane Collaboration’s tool	No	Moderate
Neelakantan P, 2019 [32]	International Endodontic Journal	2.155	Q1	Hong Kong Brazil	Yes	CT	4 (SR-MA)	Yes	Yes	Critical appraisal approach used by the Oxford Centre for Evidence Medicine Cochrane Collaboration’s tool	No	Moderate
Silva EJNL, 2019 [33]	British Dental Journal	0.602	Q2	Brazil	No	RCT	3 (SR)	No	No	Cochrane Collaboration’s tool	Yes	Moderate
Anagnostaki E, 2020 [34]	Dentistry Journal (Basel)	0.564	Q2	United Kingdom United States	No	RCT	17 (SR)	Yes	Yes	Cochrane Collaboration’s tool	No	Moderate
Ruksakiet K, 2020 [35]	Journal of Endodontics	1.356	Q1	Hungary	Yes	RCT	8 (SR) 6 (MA)	Yes	Yes	Cochrane Collaboration’s tool	Yes	High
Ali NT, 2022 [36]	Odontology	0.637	Q1	Egypt	Yes	RCT	10 (SR) 9 (MA)	No	Yes	Cochrane Collaboration’s tool	Yes	High
Tonini R, 2022 [37]	Frontiers in Oral Health	0.694	Q1	Italy Spain Russia	No	RCT	7 (SR)	No	Yes	Cochrane Collaboration’s tool	No	High
Chalub LO, 2023 [38]	Clinical Oral Investigations	0.942	Q1	Brazil	Yes	RCT	12 (SR) 8 (MA)	Yes	Yes	Cochrane Collaboration’s tool	Yes	High
Meire MA, 2023 [39]	International Endodontic Journal	2.155	Q1	Belgium Brazil	Yes	RCT CT Cohort (Longitudinal)	14 (SR–MA)	Yes	Yes	Cochrane Collaboration’s tool	Yes	High
Quintana RM, 2023 [40]	Lasers in Medical Science	0.575	Q2	Brazil	Yes	RCT CT	8 (SR) 6 (MA)	Yes	Yes	Cochrane Collaboration’s tool	Yes	High
Weissheimer T, 2023 [41]	Restorative dentistry & endodontics	N/A	N/A	Brazil	Yes	RCT	6 (SR) 4 (MA)	No	Yes	Cochrane Collaboration’s tool	Yes	High
Gobbo LB, 2024 [42]	Evidence-Based Dentistry	0.247	Q3	Brazil	Yes	RCT	3 (SR–MA)	Yes	Yes	Cochrane Collaboration’s tool	Yes	High
Hazrati P, 2024 [43]	Journal of Lasers in Medical Sciences	0.380	Q2	Iran	No	RCT	8 (SR)	Yes	Yes	Cochrane Collaboration’s tool	No	Moderate

Abbreviations: RCT: randomized controlled trial; CT: controlled trial; SR: systematic review; MA: meta-analysis.

**Table 2:** Summary of main findings of the included SR–MAs according to the PICO question (n = 13).

First Author, Year	Main Descriptive Findings According to PICO Question		
	Antimicrobial Parameters	Clinical Parameters	Radiographic Parameters
Moreira RN, 2019 [31]	Similar reduction in microorganisms and/or biofilm in CI and PUI	Not evaluated	Not evaluated
Neelakantan P, 2019 [32]	NaOCl and CHX reduced the endotoxin levels compared to the initial outcomes found in primary endodontic infections.	Not evaluated	Not evaluated
Silva EJNL, 2019 [33]	Similar reduction in microorganisms and/or biofilm in CI and PUI	Not evaluated	Similar radiographic healing in CI and PUI
Anagnostaki E, 2020 [34]	Laser use increases antimicrobial efficacy in conjunction with laser use	Laser use contributes to pain reduction	Laser use increases radiographic healing
Ruksakiet K, 2020 [35]	Similar reduction in microorganisms and/or biofilm in CI and PUI	Studies did not report the disappearance of clinical symptoms	Similar radiographic healing in CI and PUI
Ali NT, 2022 [36]	Similar reduction in microorganisms and/or biofilm in CI and PUI	Similar effects regarding pain intensity and the incidence of rescue-analgesic intake and treatment failure	Not evaluated
Tonini R, 2022 [37]	Higher biofilm reduction and antimicrobial effects especially for activation methods (PUI)	Some studies reported the disappearance of clinical symptoms	One included primary study showed radiographic healing
Chalub LO, 2023 [38]	Higher antimicrobial effects especially for activation methods (PUI)	Not evaluated	Not evaluated
Meire MA, 2023 [39]	Not evaluated	No significant difference in the prevalence of pain 7-day post-treatment was demonstrated when diode laser irradiation and no adjunct therapy are compared	Similar radiographic healing in diode laser irradiation and adjunct therapy
Quintana RM, 2023 [40]	Higher antimicrobial efficacy after aPDT than CD alone	Not evaluated	Not evaluated
Weissheimer T, 2023 [41]	Similar disinfectant ability of CHX gel and NaOCl, but further research is necessary	Not evaluated	Not evaluated
Gobbo LB, 2024 [42]	Not evaluated	Higher periapical healing outcomes in PUI compared with CSI	Higher percentage of periapical healing rate in PUI compared to CSI
Hazrati P, 2024 [43]	Not evaluated	Clinical healing in conjunction with laser use, but without significant differences with traditional protocols	Radiographic healing in conjunction with laser use, but without significant differences with traditional protocols

Abbreviations: aPDT: antimicrobial photodynamic therapy; CD: chemomechanical root canal disinfection PUI: passive ultrasonic irrigation; CSI/CI: conventional syringe irrigation.

**Table 3:** Limitations of the included SR–MA.s.

Limitations	n	References
**Methodological considerations in the included primary studies**		
Microbiological assessment considering only the main root canal (or unirradicular teeth)	7	Moreira RN, 2019 [31] Silva EJNL, 2019 [33] Ruksakiet K, 2020 [35] Tonini R, 2022 [37] Chalub LO, 2023 [38] Quintana RM, 2023 [40] Weissheimer T, 2023 [41]
Lack of standardization in techniques, materials, and the concentration of the different irrigants used in endodontic irrigation protocols	7	Neelakantan P, 2019 [32] Ruksakiet K, 2020 [35] Tonini R, 2022 [37] Chalub LO, 2023 [38] Weissheimer T, 2023 [41] Gobbo LB, 2024 [42] Hazrati P, 2024 [43]
Methodological aspects related to the size and type of sample used in primary studies	6	Moreira RN, 2019 [31] Neelakantan P, 2019 [32] Silva EJNL, 2019 [33] Ruksakiet K, 2020 [35] Weissheimer T, 2023 [41] Gobbo LB, 2024 [42]
Microbiological assessment using paper points	3	Silva EJNL, 2019 [33] Ruksakiet K, 2020 [35] Tonini R, 2022 [37]
The time of application and/or other characteristics of the irrigant considering the methodological technique is not considered	3	Ruksakiet K, 2020 [35] Tonini R, 2022 [37] Gobbo LB, 2024 [42]
Lack of information about the laser usage parameters	2	Anagnostaki E, 2020 [34] Quintana RM, 2023 [40]
Different methods for quantification of lipopolysaccharides (LPS)	1	Neelakantan P, 2019 [32]
The microbiome is not considered in thirds	1	Chalub LO, 2023 [38]
The non-inclusion of teeth with complex anatomies in primary studies	1	Chalub LO, 2023 [38]
**Quality of the scientific evidence**		
Substantial heterogeneity of the included primary studies	6	Silva EJNL, 2019 [33] Ruksakiet K, 2020 [35] Ali NT, 2022 [36] Chalub LO, 2023 [38] Meire MA, 2023 [39] Weissheimer, 2023 [41]
Amount of available scientific evidence (primary studies)	5	Silva EJNL, 2019 [33] Ali NT, 2022 [36] Meire MA, 2023 [39] Weissheimer T, 2023 [41] Gobbo LB, 2024 [42]
High/moderate risk of bias of the included primary studies	2	Moreira RN, 2019 [31] Ali NT, 2022 [36]

## Supplementary Materials

The following supporting information can be downloaded at: https://www.mdpi.com/article/10.3390/dj13060273/s1, Table S1. Keywords and search strategy used in databases according to PICO(S) question; Table S2. Distribution of AMSTAR 2 item-per-item responses for the included SR–MAs; Table S3. Detailed findings of the SR–MAs included according to the PICO(S) question (n = 13) [31,32,33,34,35,36,37,38,39,40,41,42,43]; Table S4. Detailed characteristics of the irrigants and the protocols used in endodontic therapy according to the reported findings [31,32,33,34,35,36,37,38,39,40,41,42,43]; Table S5. Primary study overlap in the included SR-MAs (n = 74) [21,31,32,33,34,35,36,37,38,39,40,41,42,43,45,46,47,48,49,50,51,52,53,54,55,56,57,58,59,60,61,62,63,64,65,66,67,68,69,70,71,72,73,74,75,76,77,78,79,80,81,82,83,84,85,86,87,88,89,90,91,92,93,94,95,96,97,98,99,100,101,102,103,104,105,106,107,108,109,110,111,112,113,114,115,116,117].

## References

[1] Schilder H. (1974). Cleaning and shaping the root canal. Dent. Clin. North. Am..

[2] Peters O.A., Laib A., Göhring T.N., Barbakow F. (2001). Changes in root canal geometry after preparation assessed by high-resolution computed tomography. J. Endod..

[3] Boutsioukis C., Versiani M.A., Basrani B., Sousa-Neto M.D. (2019). Internal Tooth Anatomy and Root Canal Irrigation. The Root Canal Anatomy in Permanent Dentition.

[4] Cobankara F.K., Ozkan H.B., Terlemez A. (2010). Comparison of organic tissue dissolution capacities of sodium hypochlorite and chlorine dioxide. J. Endod..

[5] Jungbluth H., Marending M., De-Deus G., Sener B., Zehnder M. (2011). Stabilizing sodium hypochlorite at high pH: Effects on soft tissue and dentin. J. Endod..

[6] Liolios E., Economides N., Parissis-Messimeris S., Boutsioukis A. (1997). The effectiveness of three irrigating solutions on root canal cleaning after hand and mechanical preparation. Int. Endod. J..

[7] Petridis X., Busanello F.H., So M.V.R., Dijkstra R.J.B., Sharma P.K., van der Sluis L.W.M. (2019). Factors affecting the chemical efficacy of 2% sodium hypochlorite against oral steady-state dual-species biofilms: Exposure time and volume application. Int. Endod. J..

[8] Arias-Moliz M.T., Ferrer-Luque C.M., Espigares-García M., Baca P. (2009). Enterococcus faecalis biofilms eradication by root canal irrigants. J. Endod..

[9] Wang Z., Shen Y., Haapasalo M. (2020). Dynamics of Dissolution, Killing, and Inhibition of Dental Plaque Biofilm. Front. Microbiol..

[10] Du T., Wang Z., Shen Y., Ma J., Cao Y., Haapasalo M. (2014). Effect of long-term exposure to endodontic disinfecting solutions on young and old Enterococcus faecalis biofilms in dentin canals. J. Endod..

[11] Heling I., Steinberg D., Kenig S., Gavrilovich I., Sela M.N., Friedman M. (1992). Efficacy of a sustained-release device containing chlorhexidine and Ca(OH)_2_ in preventing secondary infection of dentinal tubules. Int. Endod. J..

[12] Poppolo Deus F., Ouanounou A. (2022). Chlorhexidine in Dentistry: Pharmacology, Uses, and Adverse Effects. Int. Dent. J..

[13] De-Deus G., Zehnder M., Reis C., Fidel S., Fidel R.A., Galan J., Paciornik S. (2008). Longitudinal co-site optical microscopy study on the chelating ability of etidronate and EDTA using a comparative single-tooth model. J. Endod..

[14] George S., Kishen A., Song K.P. (2005). The role of environmental changes on monospecies biofilm formation on root canal wall by Enterococcus faecalis. J. Endod..

[15] Souza R.A.d., Castro F.P.L.d., Pires O.J. (2022). Research of the major methods and clinical outcomes of irrigation in endodontics: A systematic review. MedNEXT J. Med. Health Sci..

[16] Busanello F.H., Petridis X., So M.V.R., Dijkstra R.J.B., Sharma P.K., van der Sluis L.W.M. (2019). Chemical biofilm removal capacity of endodontic irrigants as a function of biofilm structure: Optical coherence tomography, confocal microscopy and viscoelasticity determination as integrated assessment tools. Int. Endod. J..

[17] Zehnder M., Schmidlin P., Sener B., Waltimo T. (2005). Chelation in root canal therapy reconsidered. J. Endod..

[18] Hottel T.L., el-Refai N.Y., Jones J.J. (1999). A comparison of the effects of three chelating agents on the root canals of extracted human teeth. J. Endod..

[19] Park E., Shen Y., Haapasalo M. (2012). Irrigation of the apical root canal. Endod. Topics.

[20] Boutsioukis C., Arias-Moliz M.T. (2022). Present status and future directions—Irrigants and irrigation methods. Int. Endod. J..

[21] Huffaker S.K., Safavi K., Spangberg L.S., Kaufman B. (2010). Influence of a passive sonic irrigation system on the elimination of bacteria from root canal systems: A clinical study. J. Endod..

[22] Kasić S., Knezović M., Beader N., Gabrić D., Malčić A.I., Baraba A. (2017). Efficacy of Three Different Lasers on Eradication of Enterococcus faecalis and Candida albicans Biofilms in Root Canal System. Photomed. Laser Surg..

[23] Aromataris E., Fernandez R., Godfrey C.M., Holly C., Khalil H., Tungpunkom P. (2015). Summarizing systematic reviews: Methodological development, conduct and reporting of an umbrella review approach. Int. J. Evid. Based Healthc..

[24] Page M.J., McKenzie J.E., Bossuyt P.M., Boutron I., Hoffmann T.C., Mulrow C.D., Shamseer L., Tetzlaff J.M., Akl E.A., Brennan S.E. (2021). The PRISMA 2020 statement: An updated guideline for reporting systematic reviews. BMJ.

[25] Higgins J., Thomas J., Chandler J., Cumpston M., Li T., Page M., Welch V. (2019). Cochrane Handbook for Systematic Reviews of Interventions.

[26] Centre for Reviews and Dissemination (CRD)—University of York CDR Database. https://www.york.ac.uk/crd/.

[27] Shea B.J., Reeves B.C., Wells G., Thuku M., Hamel C., Moran J., Moher D., Tugwell P., Welch V., Kristjansson E. (2017). AMSTAR 2: A critical appraisal tool for systematic reviews that include randomised or non-randomised studies of healthcare interventions, or both. BMJ.

[28] Pieper D., Antoine S.L., Mathes T., Neugebauer E.A., Eikermann M. (2014). Systematic review finds overlapping reviews were not mentioned in every other overview. J. Clin. Epidemiol..

[29] Kirvalidze M., Abbadi A., Dahlberg L., Sacco L.B., Calderón-Larrañaga A., Morin L. (2023). Estimating pairwise overlap in umbrella reviews: Considerations for using the corrected covered area (CCA) index methodology. Res. Synth. Methods.

[30] Higgins J.P., Thompson S.G., Deeks J.J., Altman D.G. (2003). Measuring inconsistency in meta-analyses. BMJ.

[31] Moreira R.N., Pinto E.B., Galo R., Falci S.G.M., Mesquita A.T. (2019). Passive ultrasonic irrigation in root canal: Systematic review and meta-analysis. Acta Odontol. Scand..

[32] Neelakantan P., Herrera D.R., Pecorari V.G.A., Gomes B. (2019). Endotoxin levels after chemomechanical preparation of root canals with sodium hypochlorite or chlorhexidine: A systematic review of clinical trials and meta-analysis. Int. Endod. J..

[33] Silva E., Rover G., Belladonna F.G., Herrera D.R., De-Deus G., da Silva Fidalgo T.K. (2019). Effectiveness of passive ultrasonic irrigation on periapical healing and root canal disinfection: A systematic review. Br. Dent. J..

[34] Anagnostaki E., Mylona V., Parker S., Lynch E., Grootveld M. (2020). Systematic Review on the Role of Lasers in Endodontic Therapy: Valuable Adjunct Treatment?. Dent. J..

[35] Ruksakiet K., Hanák L., Farkas N., Hegyi P., Sadaeng W., Czumbel L.M., Sang-Ngoen T., Garami A., Mikó A., Varga G. (2020). Antimicrobial Efficacy of Chlorhexidine and Sodium Hypochlorite in Root Canal Disinfection: A Systematic Review and Meta-analysis of Randomized Controlled Trials. J. Endod..

[36] Ali N.T., El-Boghdadi R.M., Ibrahim A.M., Amin S.A.W. (2022). Clinical and microbiological effects of ultrasonically activated irrigation versus syringe irrigation during endodontic treatment: A systematic review and meta-analysis of randomized clinical trials. Odontology.

[37] Tonini R., Salvadori M., Audino E., Sauro S., Garo M.L., Salgarello S. (2022). Irrigating Solutions and Activation Methods Used in Clinical Endodontics: A Systematic Review. Front. Oral. Health.

[38] Chalub L.O., Nunes G.P., Strazzi-Sahyon H.B., Ferrisse T.M., Dos Santos P.H., Gomes-Filho J.E., Cintra L.T.A., Sivieri-Araujo G. (2023). Antimicrobial effectiveness of ultrasonic irrigation in root canal treatment: A systematic review of randomized clinical trials and meta-analysis. Clin. Oral. Investig..

[39] Meire M.A., Bronzato J.D., Bomfim R.A., Gomes B. (2023). Effectiveness of adjunct therapy for the treatment of apical periodontitis: A systematic review and meta-analysis. Int. Endod. J..

[40] Quintana R.M., Scarparo R.K., Münchow E.A., Pinheiro L.S., Tavares C.O., Kopper P.M.P. (2023). Does aPDT reduce bacterial load in endodontic infected teeth? A systematic review and meta-analysis. Lasers Med. Sci..

[41] Weissheimer T., Pinto K.P., da Silva E., Hashizume L.N., da Rosa R.A., Só M.V.R. (2023). Disinfectant effectiveness of chlorhexidine gel compared to sodium hypochlorite: A systematic review with meta-analysis. Restor. Dent. Endod..

[42] Gobbo L.B., de Araújo L.P., Vieira W.A., de-Jesus-Soares A., de Almeida J.F.A., Ferraz C.C.R. (2024). Impact of passive ultrasonic irrigation on the outcome of non-surgical root canal treatment: A systematic review and meta-analysis of randomized clinical trials. Evid. Based Dent..

[43] Hazrati P., Azadi A., Tizno A., Asnaashari M. (2024). The Effect of Lasers on the Healing of Periapical Lesion: A Systematic Review. J. Lasers Med. Sci..

[44] Schünemann H.J. (2022). Using systematic reviews in guideline development: The GRADE approach.S ystematic Reviews in Health Research: Meta-Analysis in Context. Res. Synth. Methods.

[45] Liang Y.H., Jiang L.M., Jiang L., Chen X.B., Liu Y.Y., Tian F.C., Bao X.D., Gao X.J., Versluis M., Wu M.K. (2013). Radiographic healing after a root canal treatment performed in single-rooted teeth with and without ultrasonic activation of the irrigant: A randomized controlled trial. J. Endod..

[46] Xavier A.C., Martinho F.C., Chung A., Oliveira L.D., Jorge A.O., Valera M.C., Carvalho C.A. (2013). One-visit versus two-visit root canal treatment: Effectiveness in the removal of endotoxins and cultivable bacteria. J. Endod..

[47] Nakamura V.C., Pinheiro E.T., Prado L.C., Silveira A.C., Carvalho A.P.L., Mayer M.P.A., Gavini G. (2018). Effect of ultrasonic activation on the reduction of bacteria and endotoxins in root canals: A randomized clinical trial. Int. Endod. J..

[48] Morsy D.A., Negm M., Diab A., Ahmed G. (2018). Postoperative pain and antibacterial effect of 980 nm diode laser versus conventional endodontic treatment in necrotic teeth with chronic periapical lesions: A randomized control trial. F1000Research.

[49] Tang Z., Wang H., Jiang S. (2015). Clinical study of single-visit root canal treatment with a nickel-titanium (Ni-Ti) rotary instrument combined with different ultrasonic irrigation solutions for elderly patients with chronic apical periodontitis. Biomed. Mater. Eng..

[50] Orozco E.I.F., Toia C.C., Cavalli D., Khoury R.D., Cardoso F., Bresciani E., Valera M.C. (2020). Effect of passive ultrasonic activation on microorganisms in primary root canal infection: A randomized clinical trial. J. Appl. Oral. Sci..

[51] Ballal N.V., Gandhi P., Shenoy P.A., Dummer P.M.H. (2020). Evaluation of various irrigation activation systems to eliminate bacteria from the root canal system: A randomized controlled single blinded trial. J. Dent..

[52] Carver K., Nusstein J., Reader A., Beck M. (2007). In vivo antibacterial efficacy of ultrasound after hand and rotary instrumentation in human mandibular molars. J. Endod..

[53] Beus C., Safavi K., Stratton J., Kaufman B. (2012). Comparison of the effect of two endodontic irrigation protocols on the elimination of bacteria from root canal system: A prospective, randomized clinical trial. J. Endod..

[54] Gomes B.P., Martinho F.C., Vianna M.E. (2009). Comparison of 2.5% sodium hypochlorite and 2% chlorhexidine gel on oral bacterial lipopolysaccharide reduction from primarily infected root canals. J. Endod..

[55] Marinho A.C., Martinho F.C., Zaia A.A., Ferraz C.C., Gomes B.P. (2014). Monitoring the effectiveness of root canal procedures on endotoxin levels found in teeth with chronic apical periodontitis. J. Appl. Oral. Sci..

[56] Marinho A.C., Martinho F.C., Leite F.R., Nascimento G.G., Gomes B.P. (2015). Proinflammatory Activity of Primarily Infected Endodontic Content against Macrophages after Different Phases of the Root Canal Therapy. J. Endod..

[57] Vianna M.E., Horz H.P., Gomes B.P., Conrads G. (2006). In vivo evaluation of microbial reduction after chemo-mechanical preparation of human root canals containing necrotic pulp tissue. Int. Endod. J..

[58] Zandi H., Rodrigues R.C., Kristoffersen A.K., Enersen M., Mdala I., Ørstavik D., Rôças I.N., Siqueira J.F. (2016). Antibacterial Effectiveness of 2 Root Canal Irrigants in Root-filled Teeth with Infection: A Randomized Clinical Trial. J. Endod..

[59] Johnson K.L. (2011). A Comparison of the Effectiveness of Three Irrigation Methods in the Removal of Bacteria from Root Canals Following Instrumentation. Master’s Thesis.

[60] Middha M., Sangwan P., Tewari S., Duhan J. (2017). Effect of continuous ultrasonic irrigation on postoperative pain in mandibular molars with nonvital pulps: A randomized clinical trial. Int. Endod. J..

[61] Aveiro E., Chiarelli-Neto V.M., de-Jesus-Soares A., Zaia A.A., Ferraz C.C.R., Almeida J.F.A., Marciano M.A., Feres M., Gomes B. (2020). Efficacy of reciprocating and ultrasonic activation of 6% sodium hypochlorite in the reduction of microbial content and virulence factors in teeth with primary endodontic infection. Int. Endod. J..

[62] Koba K., Kimura Y., Matsumoto K., Watanabe H., Shinoki T., Kojy R., Ito M. (1999). Post-operative symptoms and healing after endodontic treatment of infected teeth using pulsed Nd:YAG laser. Endod. Dent. Traumatol..

[63] Masilionyte M., Gutknecht N. (2018). Outcome of 940-nm diode laser-assisted endodontic treatment of teeth with apical periodontitis: A retrospective study of clinical cases. Laser Dent. Sci..

[64] Martins M.R., Carvalho M.F., Pina-Vaz I., Capelas J.A., Martins M.A., Gutknecht N. (2014). Outcome of Er,Cr:YSGG laser-assisted treatment of teeth with apical periodontitis: A blind randomized clinical trial. Photomed. Laser Surg..

[65] Verma A., Yadav R.K., Tikku A.P., Chandra A., Verma P., Bharti R., Shakya V.K. (2020). A randomized controlled trial of endodontic treatment using ultrasonic irrigation and laser activated irrigation to evaluate healing in chronic apical periodontitis. J. Clin. Exp. Dent..

[66] Garcez A., Núñez S., Hamblin M., Ribeiro M. (2008). Antimicrobial Comparison on Effectiveness of Endodontic Therapy and Endodontic Therapy Combined with Photo-Disinfection on Patients with Periapical Lesion: A 6 Month Follow-Up.

[67] Rôças I.N., Provenzano J.C., Neves M.A., Siqueira J.F. (2016). Disinfecting Effects of Rotary Instrumentation with Either 2.5% Sodium Hypochlorite or 2% Chlorhexidine as the Main Irrigant: A Randomized Clinical Study. J. Endod..

[68] Burleson A., Nusstein J., Reader A., Beck M. (2007). The in vivo evaluation of hand/rotary/ultrasound instrumentation in necrotic, human mandibular molars. J. Endod..

[69] Paiva S.S., Siqueira J.F., Rôças I.N., Carmo F.L., Ferreira D.C., Curvelo J.A., Soares R.M., Rosado A.S. (2012). Supplementing the antimicrobial effects of chemomechanical debridement with either passive ultrasonic irrigation or a final rinse with chlorhexidine: A clinical study. J. Endod..

[70] Paiva S.S., Siqueira J.F., Rôças I.N., Carmo F.L., Leite D.C., Ferreira D.C., Rachid C.T., Rosado A.S. (2013). Molecular microbiological evaluation of passive ultrasonic activation as a supplementary disinfecting step: A clinical study. J. Endod..

[71] Herrera D.R., Martinho F.C., de-Jesus-Soares A., Zaia A.A., Ferraz C.C.R., Almeida J.F.A., Gomes B. (2017). Clinical efficacy of EDTA ultrasonic activation in the reduction of endotoxins and cultivable bacteria. Int. Endod. J..

[72] Garcez A.S., Nuñez S.C., Hamblim M.R., Suzuki H., Ribeiro M.S. (2010). Photodynamic therapy associated with conventional endodontic treatment in patients with antibiotic-resistant microflora: A preliminary report. J. Endod..

[73] Jurič I.B., Plečko V., Pandurić D.G., Anić I. (2014). The antimicrobial effectiveness of photodynamic therapy used as an addition to the conventional endodontic re-treatment: A clinical study. Photodiagnosis. Photodyn. Ther..

[74] Yoo Y.J., Shon W.J., Baek S.H., Kang M.K., Kim H.C., Lee W. (2014). Effect of 1440-nanometer neodymium:yttrium-aluminum-garnet laser irradiation on pain and neuropeptide reduction: A randomized prospective clinical trial. J. Endod..

[75] Arslan H., Doğanay E., Karataş E., Ünlü M.A., Ahmed H.M.A. (2017). Effect of Low-level Laser Therapy on Postoperative Pain after Root Canal Retreatment: A Preliminary Placebo-controlled, Triple-blind, Randomized Clinical Trial. J. Endod..

[76] Asnaashari M., Ashraf H., Daghayeghi A.H., Mojahedi S.M., Azari-Marhabi S. (2017). Management of Post Endodontic Retreatment Pain With Low Level Laser Therapy. J. Lasers. Med. Sci..

[77] Pourhajibagher M., Ghorbanzadeh R., Parker S., Chiniforush N., Bahador A. (2017). The evaluation of cultivable microbiota profile in patients with secondary endodontic infection before and after photo-activated disinfection. Photodiagnosis. Photodyn. Ther..

[78] de Miranda R.G., Colombo A.P.V. (2018). Clinical and microbiological effectiveness of photodynamic therapy on primary endodontic infections: A 6-month randomized clinical trial. Clin. Oral. Investig..

[79] Doğanay Yıldız E., Arslan H. (2018). Effect of Low-level Laser Therapy on Postoperative Pain in Molars with Symptomatic Apical Periodontitis: A Randomized Placebo-controlled Clinical Trial. J. Endod..

[80] Nabi S., Amin K., Masoodi A., Farooq R., Purra A.R., Ahangar F.A. (2018). Effect of preoperative ibuprofen in controlling postendodontic pain with and without low-level laser therapy in single visit endodontics: A randomized clinical study. Indian. J. Dent. Res..

[81] Arslan H., Köseoğlu S., Doğanay Yildiz E., Arabaci T., Savran L., Yildiz D.A., Veyisoğlu G. (2019). Effect of intracanal diode laser application and low-level laser therapy on CGRP change. Braz. Oral. Res..

[82] Coelho M.S., Vilas-Boas L., Tawil P.Z. (2019). The effects of photodynamic therapy on postoperative pain in teeth with necrotic pulps. Photodiagnosis. Photodyn. Ther..

[83] Dagher J., El Feghali R., Parker S., Benedicenti S., Zogheib C. (2019). Postoperative Quality of Life Following Conventional Endodontic Intracanal Irrigation Compared with Laser-Activated Irrigation: A Randomized Clinical Study. Photobiomodul. Photomed. Laser. Surg..

[84] Doğanay Yıldız E., Arslan H., Köseoğlu S., Arabacı T., Yıldız D.A., Savran L. (2019). The effect of photobiomodulation on total amount of substance P in gingival crevicular fluid: Placebo-controlled randomized clinical trial. Lasers. Med. Sci..

[85] Genc Sen O., Kaya M. (2019). Effect of Root Canal Disinfection with a Diode Laser on Postoperative Pain After Endodontic Retreatment. Photobiomodul. Photomed. Laser. Surg..

[86] Lopes L.P.B., Herkrath F.J., Vianna E.C.B., Gualberto Júnior E.C., Marques A.A.F., Sponchiado Júnior E.C. (2019). Effect of photobiomodulation therapy on postoperative pain after endodontic treatment: A randomized, controlled, clinical study. Clin. Oral. Investig..

[87] Nunes E.C., Herkrath F.J., Suzuki E.H., Gualberto Júnior E.C., Marques A.A.F., Sponchiado Júnior E.C. (2020). Comparison of the effect of photobiomodulation therapy and Ibuprofen on postoperative pain after endodontic treatment: Randomized, controlled, clinical study. Lasers. Med. Sci..

[88] Ringel A.M., Patterson S.S., Newton C.W., Miller C.H., Mulhern J.M. (1982). In vivo evaluation of chlorhexidine gluconate solution and sodium hypochlorite solution as root canal irrigants. J. Endod..

[89] Kuruvilla J.R., Kamath M.P. (1998). Antimicrobial activity of 2.5% sodium hypochlorite and 0.2% chlorhexidine gluconate separately and combined, as endodontic irrigants. J. Endod..

[90] Ercan E., Ozekinci T., Atakul F., Gül K. (2004). Antibacterial activity of 2% chlorhexidine gluconate and 5.25% sodium hypochlorite in infected root canal: In vivo study. J. Endod..

[91] Zandi H., Petronijevic N., Mdala I., Kristoffersen A.K., Enersen M., Rôças I.N., Siqueira J.F., Ørstavik D. (2019). Outcome of Endodontic Retreatment Using 2 Root Canal Irrigants and Influence of Infection on Healing as Determined by a Molecular Method: A Randomized Clinical Trial. J. Endod..

[92] Jain A., Bahuguna R., Kashyap S., Ali A. (2017). Incidence of post endodontic pain after single visit root canal treatment with manual, rotary and rotary instruments with ultrasonic cleaning: A comparative study. Saudi. J. Oral. Dent. Res..

[93] Topçuoğlu H.S., Topçuoğlu G., Arslan H. (2018). The Effect of Different Irrigation Agitation Techniques on Postoperative Pain in Mandibular Molar Teeth with Symptomatic Irreversible Pulpitis: A Randomized Clinical Trial. J. Endod..

[94] Malkhassian G., Manzur A.J., Legner M., Fillery E.D., Manek S., Basrani B.R., Friedman S. (2009). Antibacterial efficacy of MTAD final rinse and two percent chlorhexidine gel medication in teeth with apical periodontitis: A randomized double-blinded clinical trial. J. Endod..

[95] Ballal N.V., Gandhi P., Shenoy P.A., Shenoy Belle V., Bhat V., Rechenberg D.K., Zehnder M. (2019). Safety assessment of an etidronate in a sodium hypochlorite solution: Randomized double-blind trial. Int. Endod. J..

[96] Bellingham A., John P. (2011). In Vivo Evaluation of Contemporary Endodontic Antimicrobial Procedures.

[97] Lu C.H., Zhong Q. (2017). Comparison of antimicrobial activity of Er,Cr: YSGG laser and ultrasonic irrigation in root canal disinfection. Shanghai Kou Qiang Yi Xue.

[98] Bilgin B., Türker S.A., Aslan M.H., Saǧlam B.C., Koçak S., Koçak M.M., Bodrumlu E. (2020). Entrococcus faecalis elimination in retreatment cases using passive ultrasonic irrigation, manual dynamic activation and photodynamic therapy: A randomized clinical trial. Tanta Dent. J..

[99] Palanisamy R. (2020). Clinical Evaluation of Post-Operative Pain after the Application of Different Irrigation Methods: An In Vivo study.

[100] Shehab Aldean A.A., Darrag A.M., Shaheen N.A., Ezzat M.M. (2020). Microbial reduction after using different root canal irrigation–activation techniques. Tanta. Dent. J..

[101] Barciela B., da Silva Limoeiro A.G., Bueno C.E., Fernandes S.L., Mandarini D.R., Boer N.C., Camara Fernandes K.G., Rocha D.G. (2019). In vivo evaluation of painful symptomatology after endodontic treatment with or without the use of photodynamic therapy. J. Conserv. Dent..

[102] Guimarães L.D.S., da Silva E.A.B., Hespanhol F.G., Fontes K., Antunes L.A.A., Antunes L.S. (2021). Effect of photobiomodulation on post-operative symptoms in teeth with asymptomatic apical periodontitis treated with foraminal enlargement: A randomized clinical trial. Int. Endod. J..

[103] Kaplan T., Sezgin G.P., Sönmez Kaplan S. (2021). Effect of a 980-nm diode laser on post-operative pain after endodontic treatment in teeth with apical periodontitis: A randomized clinical trial. BMC. Oral. Health..

[104] Kist S., Kollmuss M., Jung J., Schubert S., Hickel R., Huth K.C. (2017). Comparison of ozone gas and sodium hypochlorite/chlorhexidine two-visit disinfection protocols in treating apical periodontitis: A randomized controlled clinical trial. Clin. Oral. Investig..

[105] Souza M.A., Bonacina L.V., Trento A., Bonfante F.D.C., Porsch H.F., Ricci R., Lago B.L.T., Lago C.T.R., Gabrielli E.S., Bervian J. (2021). Influence of the apical limit of instrumentation and photodynamic therapy on the postoperative pain of lower molars with asymptomatic apical periodontitis. Photodiagnosis. Photodyn. Ther..

[106] Pietrzycka K., Pawlicka H. (2022). Effectiveness of one-visit treatment of teeth with infected root canals with and without ozonotherapy. J. Stomatol..

[107] Garcez A.S., Nuñez S.C., Hamblin M.R., Ribeiro M.S. (2008). Antimicrobial effects of photodynamic therapy on patients with necrotic pulps and periapical lesion. J. Endod..

[108] Granevik Lindström M., Wolf E., Fransson H. (2017). The Antibacterial Effect of Nd:YAG Laser Treatment of Teeth with Apical Periodontitis: A Randomized Controlled Trial. J. Endod..

[109] Rabello D.G.D., Corazza B.J.M., Ferreira L.L., Santamaria M.P., Gomes A.P.M., Martinho F.C. (2017). Does supplemental photodynamic therapy optimize the disinfection of bacteria and endotoxins in one-visit and two-visit root canal therapy? A randomized clinical trial. Photodiagnosis. Photodyn. Ther..

[110] Razumova S., Brago A., Barakat H., Howijieh A., Senyagin A., Serebrov D., Guryeva Z., Kozlova Y., Adzhieva E. (2022). Evaluation of the Microbiological Effect of Colloidal Nanosilver Solution for Root Canal Treatment. J. Funct. Biomater.

[111] Zorita-García M., Alonso-Ezpeleta L., Cobo M., Del Campo R., Rico-Romano C., Mena-Álvarez J., Zubizarreta-Macho Á. (2019). Photodynamic therapy in endodontic root canal treatment significantly increases bacterial clearance, preventing apical periodontitis. Quintessence. Int..

[112] Di Taranto V.D., Libonati A., Montemurro E., Gallusi G., Campanella V. (2022). Antimicrobial effects of Photodynamic and high-power laser endodontic therapy on patients with necrotic pulp and periapical lesion. J. Biol. Regul. Homeost. Agents.

[113] Barbosa-Ribeiro M., De-Jesus-Soares A., Zaia A.A., Ferraz C.C., Almeida J.F., Gomes B.P. (2016). Quantification of Lipoteichoic Acid Contents and Cultivable Bacteria at the Different Phases of the Endodontic Retreatment. J. Endod..

[114] Dalaei Moghadam M., Saberi E.A., Farhad Molashahi N., Shahraki Ebrahimi H. (2021). Comparative efficacy of depotphoresis and diode laser for reduction of microbial load and postoperative pain, and healing of periapical lesions: A randomized clinical trial. G. Ital. Endod..

[115] Shaheed A.A., Jawad H.A., Hussain B.M., Said A.M. (2020). Healing of apical periodontitis after minimally invasive endodontics therapy using Er,Cr:YSGG laser: A prospective clinical study. Sys. Rev. Pharm..

[116] Martins M.R., Carvalho M.F., Vaz I.P., Capelas J.A., Martins M.A., Gutknecht N. (2013). Efficacy of Er,Cr:YSGG laser with endodontical radial firing tips on the outcome of endodontic treatment: Blind randomized controlled clinical trial with six-month evaluation. Lasers. Med. Sci..

[117] Karakov K.G., Gandylyan K.S., Khachaturyan E.E., Vlasova T.N., Oganyan A.V., Eremenko A.V. (2018). Comparative Characteristics of the Methods of Treatment of Chronic Periodontitis Using Antibacterial Photodynamic Therapy (Per One Visit) and Calasept Preparation. J. Natl. Med. Assoc..

[118] Kanagasingam S., Blum I.R. (2020). Sodium Hypochlorite Extrusion Accidents: Management and Medico-Legal Considerations. Prim. Dent. J..

[119] Retamozo B., Shabahang S., Johnson N., Aprecio R.M., Torabinejad M. (2010). Minimum contact time and concentration of sodium hypochlorite required to eliminate Enterococcus faecalis. J. Endod..

[120] Cai C., Chen X., Li Y., Jiang Q. (2023). Advances in the Role of Sodium Hypochlorite Irrigant in Chemical Preparation of Root Canal Treatment. Biomed. Res. Int..

[121] Rosenthal S., Spångberg L., Safavi K. (2004). Chlorhexidine substantivity in root canal dentin. Oral. Surg. Oral. Med. Oral. Pathol. Oral. Radiol. Endod..

[122] Yesilsoy C., Whitaker E., Cleveland D., Phillips E., Trope M. (1995). Antimicrobial and toxic effects of established and potential root canal irrigants. J. Endod..

[123] Berber V.B., Gomes B.P., Sena N.T., Vianna M.E., Ferraz C.C., Zaia A.A., Souza-Filho F.J. (2006). Efficacy of various concentrations of NaOCl and instrumentation techniques in reducing Enterococcus faecalis within root canals and dentinal tubules. Int. Endod. J..

[124] Siqueira J.F., Rôças I.N. (2008). Clinical implications and microbiology of bacterial persistence after treatment procedures. J. Endod..

[125] Maezono H., Klanliang K., Shimaoka T., Asahi Y., Takahashi Y., Wang Z., Shen Y., Haapasalo M., Hayashi M. (2024). Effects of Sodium Hypochlorite Concentration and Application Time on Bacteria in an Ex Vivo Polymicrobial Biofilm Model. J. Endod..

[126] Siqueira J.F., Rôças I.N. (2022). Present status and future directions: Microbiology of endodontic infections. Int. Endod. J..

[127] Álvarez-Sagües A., Herce N., Amador U., Llinares-Pinel F., Nistal-Villan E., Presa J., Álvarez L., Azabal M. (2021). Efficacy of EDTA and HEDP Chelators in the Removal of Mature Biofilm of Enterococcus faecalis by PUI and XPF File Activation. Dent. J..

[128] Mello I., Robazza C.R., Antoniazzi J.H., Coil J. (2008). Influence of different volumes of EDTA for final rinse on smear layer removal. Oral. Surg. Oral. Med. Oral. Pathol. Oral. Radiol. Endod..

[129] Mello I., Kammerer B.A., Yoshimoto D., Macedo M.C., Antoniazzi J.H. (2010). Influence of final rinse technique on ability of ethylenediaminetetraacetic acid of removing smear layer. J. Endod..

[130] Tartari T., Bachmann L., Zancan R.F., Vivan R.R., Duarte M.A.H., Bramante C.M. (2018). Analysis of the effects of several decalcifying agents alone and in combination with sodium hypochlorite on the chemical composition of dentine. Int. Endod. J..

[131] Nikhil V., Jaiswal S., Bansal P., Arora R., Raj S., Malhotra P. (2016). Effect of phytic acid, ethylenediaminetetraacetic acid, and chitosan solutions on microhardness of the human radicular dentin. J. Conserv. Dent..

[132] Bosaid F., Aksel H., Makowka S., Azim A.A. (2020). Surface and structural changes in root dentine by various chelating solutions used in regenerative endodontics. Int. Endod. J..

[133] Darda S., Madria K., Jamenis R., Heda A., Khanna A., Sardar L. (2014). An in-vitro evaluation of effect of EDTAC on root dentin with respect to time. J. Int. Oral. Health.

[134] Biel P., Mohn D., Attin T., Zehnder M. (2017). Interactions between the Tetrasodium Salts of EDTA and 1-Hydroxyethane 1,1-Diphosphonic Acid with Sodium Hypochlorite Irrigants. J. Endod..

[135] Haapasalo M., Qian W., Portenier I., Waltimo T. (2007). Effects of dentin on the antimicrobial properties of endodontic medicaments. J. Endod..

[136] Jain S., Patni P.M., Jain P., Raghuwanshi S., Pandey S.H., Tripathi S., Soni A. (2023). Comparison of Dentinal Tubular Penetration of Intracanal Heated and Preheated Sodium Hypochlorite Through Different Agitation Techniques. J. Endod..

[137] Yared G., Al Asmar Ramli G. (2020). Antibacterial Ability of Sodium Hypochlorite Heated in the Canals of Infected Teeth: An Ex Vivo Study. Cureus.

[138] Iandolo A., Pisano M., Abdellatif D., Sangiovanni G., Pantaleo G., Martina S., Amato A. (2023). Smear Layer and Debris Removal from Root Canals Comparing Traditional Syringe Irrigation and 3D Cleaning: An Ex Vivo Study. J. Clin. Med..

[139] Sirtes G., Waltimo T., Schaetzle M., Zehnder M. (2005). The effects of temperature on sodium hypochlorite short-term stability, pulp dissolution capacity, and antimicrobial efficacy. J. Endod..

[140] Gulsahi K., Tirali R.E., Cehreli S.B., Karahan Z.C., Uzunoglu E., Sabuncuoglu B. (2014). The effect of temperature and contact time of sodium hypochlorite on human roots infected with Enterococcus faecalis and Candida albicans. Odontology.

[141] Siddique R., Ranjan M., Jose J., Srivastav A., Rajakeerthi R., Kamath A. (2020). Clinical Quantitative Antibacterial Potency of Garlic-Lemon Against Sodium Hypochlorite in Infected Root Canals: A Double-blinded, Randomized, Controlled Clinical Trial. J. Int. Soc. Prev. Community Dent..

[142] Neelakantan P., Jagannathan N., Nazar N. (2011). Ethnopharmacological approach in Endodontic Treatment: A focused review. Int. J. Drug Dev. & Res..

[143] Sundaram D., Narayanan R.K., Vadakkepurayil K. (2016). A Comparative Evaluation on Antimicrobial Effect of Honey, Neem Leaf Extract and Sodium Hypochlorite as Intracanal Irrigant: An Ex-Vivo Study. J. Clin. Diagn. Res..

[144] Dutta A., Kundabala M. (2014). Comparative anti-microbial efficacy of Azadirachta indica irrigant with standard endodontic irrigants: A preliminary study. J. Conserv. Dent..

[145] Hosny N.S., El Khodary S.A., El Boghdadi R.M., Shaker O.G. (2021). Effect of Neem (Azadirachta indica) versus 2.5% sodium hypochlorite as root canal irrigants on the intensity of post-operative pain and the amount of endotoxins in mandibular molars with necrotic pulps: A randomized controlled trial. Int. Endod. J..

[146] Garg P., Tyagi S.P., Sinha D.J., Singh U.P., Malik V., Maccune E.R. (2014). Comparison of antimicrobial efficacy of propolis, Morinda citrifolia, Azadirachta indica, triphala, green tea polyphenols and 5.25% sodium hypochlorite against Enterococcus fecalis biofilm. Saudi Endod. J..

[147] Susila A.V., Sai S., Sharma N., Balasubramaniam A., Veronica A.K., Nivedhitha S. (2023). Can natural irrigants replace sodium hypochlorite? A systematic review. Clin. Oral. Investig..

[148] Pandranki J., Narasimha Rao V., Vijayalakshmi G., Dorothy K. (2013). Ethnobotanical approach against resistant endodontic pathogens using Morinda species–An antimicrobial study. Int. J. Biol. Med. Res..

[149] Podar R., Kulkarni G.P., Dadu S.S., Singh S., Singh S.H. (2015). In vivo antimicrobial efficacy of 6% Morinda citrifolia, Azadirachta indica, and 3% sodium hypochlorite as root canal irrigants. Eur. J. Dent..

[150] Murray P.E., Farber R.M., Namerow K.N., Kuttler S., Garcia-Godoy F. (2008). Evaluation of Morinda citrifolia as an Endodontic Irrigant. J. Endod..

[151] Safadi S., Maan H., Kolodkin-Gal I., Tsesis I., Rosen E. (2022). The Products of Probiotic Bacteria Effectively Treat Persistent Enterococcus faecalis Biofilms. Pharmaceutics.

[152] El-Sayed H., Aly Y., Elgamily H., Nagy M.M. (2019). A Promising Probiotic Irrigant: An In Vitro Study. Open Access Maced. J. Med. Sci..

[153] Widyarman A.S., Halim L.A., Jesslyn, Irma H.A., Richi M., Rizal M.I. (2023). The potential of reuterin derived from Indonesian strain of Lactobacillus reuteri against endodontic pathogen biofilms in vitro and ex vivo. Saudi Dent. J..

[154] Gołąbek H., Borys K.M., Kohli M.R., Brus-Sawczuk K., Strużycka I. (2019). Chemical aspect of sodium hypochlorite activation in obtaining favorable outcomes of endodontic treatment: An in-vitro study. Adv. Clin. Exp. Med..

[155] Ahmad M., Pitt Ford T.J., Crum L.A. (1987). Ultrasonic debridement of root canals: Acoustic streaming and its possible role. J. Endod..

[156] Chopra S., Murray P.E., Namerow K.N. (2008). A scanning electron microscopic evaluation of the effectiveness of the F-file versus ultrasonic activation of a K-file to remove smear layer. J. Endod..

[157] Teo C.Y.J., George R., Walsh L.J. (2018). Dispersion of near-infrared laser energy through radicular dentine when using plain or conical tips. Lasers Med. Sci..

[158] De Meyer S., Meire M.A., Coenye T., De Moor R.J. (2017). Effect of laser-activated irrigation on biofilms in artificial root canals. Int. Endod. J..

[159] Garcia-Diaz M., Huang Y.Y., Hamblin M.R. (2016). Use of fluorescent probes for ROS to tease apart Type I and Type II photochemical pathways in photodynamic therapy. Methods.

