# Supplementary material for: Effectiveness of Irrigation Protocols in Endodontic Therapy: An Umbrella Review
Source: Dent J (Basel). 2025 Jun 18;13(6):273. doi: 10.3390/dj13060273 (PMC12192043; doi:10.3390/dj13060273)
Supplement: Supplementary file 1 [file dentistry-13-00273-s001.zip › dentistry-3626882-supplementary.pdf]

Table S1. Keywords and search strategy used in databases according to PICO(S) question.

<table>
<tr><th colspan="4">What is the effectiveness of the different irrigation protocols used in endodontic therapy?</th></tr>
<tr><td>Population:
Patients requiring endodontic treatment/endodontically treated teeth</td><td>Intervention/Comparison:
Different types of endodontic irrigation protocols</td><td>Outcome:
Effectiveness of the endodontic therapy (clinical, radiographical, and microbiological)</td><td>Study</td></tr>
<tr><th colspan="4">MeSH Terms (definitions provided by PubMed):</th></tr>
<tr><td rowspan="3">No keywords or MeSH term was used to define the population. This aspect was defined by means of the inclusion criteria</td><td colspan="2">• Root Canal Irrigants: Chemicals used mainly to disinfect root canals after pulpectomy and before obturation. The major ones are camphorated monochlorophenol, EDTA, formocresol, hydrogen peroxide, metacresylacetate, and sodium hypochlorite. Root canal irrigants include also rinsing solutions of distilled water, sodium chloride, etc. Year introduced: 1984
Entry terms:
Canal Irrigant, Root
Canal Irrigants, Root
Irrigant, Root Canal
Irrigants, Root Canal
Root Canal Irrigant
Root Canal Medicament
Root Canal Medicaments
Canal Medicament, Root
Canal Medicaments, Root
Medicament, Root Canal
Medicaments, Root Canal
• Dental Disinfectants: Chemicals especially for use on instruments to destroy pathogenic organisms. (Boucher, Clinical Dental Terminology, 4th ed). Year introduced: 1997
Entry terms:
Disinfectants, Dental</td><td rowspan="3">• Systematic Review [Publication Type]: A review of primary literature in health and health policy that attempts to identify, appraise, and synthesize all the empirical evidence that meets specified eligibility criteria to answer a given research question. Its conduct uses explicit methods aimed at minimizing bias in order to produce more reliable findings regarding the effects of interventions for prevention, treatment, and rehabilitation that can be used to inform decision making.
• Systematic Reviews as Topic: Works about a review of primary literature in health and health policy that attempt to identify, appraise, and synthesize all the empirical evidence that meets specified eligibility criteria to answer a given research question. It's conducted using explicit methods aimed at minimizing bias in order to produce more reliable findings regarding the effects of interventions for prevention, treatment, and rehabilitation that can be used to inform decision making.
• Meta-Analysis as Topic: A quantitative method of combining the results of independent studies (usually drawn from the published literature) and synthesizing summaries and conclusions which may be used to evaluate therapeutic effectiveness, plan new studies, etc., with application chiefly in the areas of research and medicine.</td></tr>
<tr><th colspan="2">Free terms</th></tr>
<tr><td colspan="2">endodontic irrigants
endodontic disinfection
endodontic irrigation
endodontic irrigation protocol
root canal disinfection
root canal disinfectants</td></tr>
<tr><th>Database</th><th colspan="2">Search equation</th><th>Number of records</th></tr>
</table>

**PubMed- MEDLINE**	Search: ((((((("Root Canal Irrigants"[Mesh]) OR ("Dental Disinfectants"[Mesh])) OR (endodontic irrigants)) OR (endodontic disinfection)) OR (endodontic irrigation)) OR (endodontic irrigation protocol)) OR (root canal disinfection)) OR (root canal disinfectants) Filters: Systematic Review, English, Portuguese, Spanish, Humans Sort by: Most Recent	205
**EMBASE**	(('root canal irrigation'/exp OR 'root canal irrigation' OR 'disinfectant agent' OR (endodontic AND irrigants) OR (endodontic AND disinfection) OR (endodontic AND irrigation) OR (endodontic AND irrigation AND protocol) OR (root AND canal AND disinfection) OR (root AND canal AND disinfectants)) AND ([embase]/lim NOT ([embase]/lim AND [medline]/lim) OR ([embase classic]/lim NOT ([embase classic]/lim AND [medline]/lim))) AND 'review'/it) AND 'systematic review'/de	46
**SCOPUS**	(TITLE-ABS-KEY ("Root Canal Irrigants") OR TITLE-ABS-KEY ("Dental Disinfectants") OR TITLE-ABS-KEY ("endodontic irrigants") OR TITLE-ABS-KEY ("endodontic disinfection") OR TITLE-ABS-KEY ("endodontic irrigation") OR TITLE-ABS-KEY ("endodontic irrigation protocol") OR TITLE-ABS-KEY ("root canal disinfection") OR TITLE-ABS-KEY ("root canal disinfectants")) AND (LIMIT-TO (DOCTYPE , "re"))	117
**LILACS**	(irrigantes del conducto radicular) OR (irrigación del conducto radicular) AND db:("LILACS") AND type_of_study:("systematic_reviews") AND (instance:"lilacsplus")	15
**Google Scholar**	((irrigantes del conducto radicular) OR (irrigación del conducto radicular)) AND (revisión sistemática)<br>(endodontic AND irrigants) OR (endodontic AND disinfection) OR (endodontic AND irrigation) OR (endodontic AND irrigation AND protocol) OR (root AND canal AND disinfection) OR (root AND canal AND disinfectants))	210*

*After the initial screening conducted by the authors

Table S2. Distribution of AMSTAR 2 item-per-item responses for the included SR-MAs.

AMSTAR- 2 Items	No (%)	Partial yes (%)	Yes (%)	No meta-analysis (%)
1. Did the research questions and inclusion criteria for the review include the components of PICO?	0 (0.0)	0 (0.0)	13 (100.0)	0 (0.0)
2. Did the report of the review contain an explicit statement that the review methods were established prior to the conduct of the review and did the report justify any significant deviations from the protocol?	0 (0.0)	3 (23.1)	10 (76.9)	0 (0.0)
3. Did the review authors explain their selection of the study designs for inclusion in the review?	0 (0.0)	0 (0.0)	13 (100.0)	0 (0.0)
4. Did the review authors use a comprehensive literature search strategy?	0 (0.0)	7 (53.8)	6 (46.2)	0 (0.0)
5. Did the review authors perform study selection in duplicate?	0 (0.0)	0 (0.0)	13 (100.0)	0 (0.0)
6. Did the review authors perform data extraction in duplicate?	1 (7.7)	0 (0.0)	12 (92.3)	0 (0.0)
7. Did the review authors provide a list of excluded studies and justify the exclusions?	0 (0.0)	0 (0.0)	13 (100.0)	0 (0.0)
8. Did the review authors describe the included studies in adequate detail?	0 (0.0)	0 (0.0)	13 (100.0)	0 (0.0)
9. Did the review authors use a satisfactory technique for assessing the risk of bias (RoB) in individual studies that were included in the review?	0 (0.0)	0 (0.0)	13 (100.0)	0 (0.0)
10. Did the review authors report on the sources of funding for the studies included in the review?	11 (84.6)	0 (0.0)	2 (15.4)	0 (0.0)
11. If meta-analysis was performed did the review authors use appropriate methods for statistical combination of results?	0 (0.0)	0 (0.0)	9 (69.2)	4 (30.8)
12. If meta-analysis was performed, did the review authors assess the potential impact of RoB in individual studies on the results of the meta-analysis or other evidence synthesis?	0 (0.0)	0 (0.0)	9 (69.2)	4 (30.8)
13. Did the review authors account for RoB in individual studies when interpreting/ discussing the results of the review?	0 (0.0)	1 (7.7)	12 (92.3)	0 (0.0)
14. Did the review authors provide a satisfactory explanation for, and discussion of, any heterogeneity observed in the results of the review?	3 (23.1)	1 (7.7)	9 (69.2)	0 (0.0)
15. If they performed quantitative synthesis did the review authors carry out an adequate investigation of publication bias (small study bias) and discuss its likely impact on the results of the review?	1 (7.7)	0 (0.0)	8 (61.5)	4 (30.8)
16. Did the review authors report any potential sources of conflict of interest, including any funding they received for conducting the review?	1 (7.7)	0 (0.0)	12 (92.3)	0 (0.0)

Table S3. Detailed findings of the SR-MAs included according to the PICO(S) question (n=13) [31-43].

First author, year	Objective	Question	Population	Intervention	Comparison	Outcome	Main descriptive findings according to PICO Question			
							**Antimicrobial efficacy**	**Clinical efficacy**	**Radiographic parameters**	**Quality of evidence according to the quality assessment**
Moreira RN, 2019 [31]	To investigate whether there are differences between the root canal disinfection, comparing the passive ultrasonic irrigation technique with the conventional technique.	Reported as objective	Patients submitted to endodontic treatment	Conventional Irrigation	Passive ultrasonic irrigation	Bacterial infection	According to the MA, there was no statistical difference between the groups (although two studies showed better performance in the PUI	Not evaluated	Not evaluated	Low (2 studies with uncertain risk of bias, 2 studies with high risk)
Neelakantan P, 2019 [32]	Reported as question	In patients with primary endodontic infection, is there a statistically significant difference in the endotoxin levels after chemomechanical preparation with NaOCl or CHX?	Patients with the need of endodontic treatment due to root canal infection in permanent teeth	Chemomechanical preparation during endodontic treatment	Chemical auxiliary substances (NaOCl and CHX) used during chemomechanical preparation	Reduction of endotoxin	Chemomechanical canal preparation with both, NaOCl and CHX, reduced the endotoxin levels compared to the initial ones found in primary endodontic infections.	Not evaluated	Not evaluated	Low (one study)

Silva EJNL, 2019 [33]	To evaluate the effectiveness of passive ultrasonic irrigation (PUI) compared with non-activated irrigation (NAI) on periapical healing and root canal disinfection	Does the use of passive ultrasonic irrigation provide better healing and disinfection when compared to non-activated irrigating?	Adult patients with fully formed permanent teeth undergoing endodontic treatment	Passive ultrasonic irrigation protocol	Non-activated irrigation protocol	Endodontic treatment outcome (periapical healing)	Inconclusive: findings not showed better performance of PUI	Not evaluated	Inconclusive: findings not showed better performance of PUI	High (all studies with low risk of bias)
Anagnostaki E, 2020 [34]	To evaluate the evidence in clinical use within these three areas of therapy	Reported as objective	Patients submitted to endodontic treatment	Laser-assisted endodontic therapy	Laser-assisted endodontic therapy	Missing parameters of their protocols	Antimicrobial efficacy in conjunction with laser use	Pain reduction in conjunction with laser use	Radiographic healing in conjunction with laser use	Between moderate and high considering the levels of bias reported in the studies
Ruksakiet K, 2020 [35]	To compare the antimicrobial efficacy of chlorhexidine (CHX) and sodium hypochlorite (NaOCl), 2 irrigants routinely used in root canal therapy of permanent teeth	Does Chlorhexidine have more antimicrobial efficacy than Sodium hypochlorite?	Participants with pulpal and/or periapical disease who received endodontic treatment in permanent teeth	CHX irrigant	NaOCl irrigant	Primary: Reduction in the bacterial abundance and incidence of positive bacterial samples after irrigation Secondary: Improvement of clinical symptoms, periapical tissue healing, and postoperative pain	Both irrigants showed similar antimicrobial effects	Studies did not report the disappearance of clinical symptoms	One included study showed success during endodontic treatment	Low (four studies show uncertain risk of bias)

Ali NT, 2022 [36]	To systematically review clinical and microbiology-related effects of ultrasonically activated irrigation (UAI) compared to syringe irrigation (SI) during endodontic treatment.	In adults with mature permanent teeth undergoing non-surgical root canal treatment or retreatment, what is the effect of ultrasonically activated irrigation (UAI) compared to syringe irrigation (SI) on clinical (e.g. postoperative pain, analgesic intake, periapical healing) and microbiology-related (e.g. effects on microbes and/or their virulence factors such as LPS, LTA) outcomes?	Adults with mature permanent teeth undergoing non-surgical root canal treatment or retreatment	Ultrasonically activated irrigation (UAI)	Syringe irrigation (SI)	Clinical and microbiological findings	Both systems showed antimicrobial effects	Both systems had similar effects regarding pain intensity, and the incidence of rescue-analgesic intake and treatment failure	Not evaluated	Low (most of the studies showed high and uncertain risk of bias)
Tonini R, 2022 [37]	To evaluate the clinical endodontic protocols and limitations of irrigating solutions in the disinfection of the root canal system in patients with apical periodontitis (AP)	(1) What is the antibacterial effectiveness of the current irrigating solutions in the root canal system disinfection? (2) What is the antibacterial effectiveness of the current irrigation activation systems in the root canal system disinfection?	Patients or teeth with AP	Irrigating solutions (NaOCl, EDTA, CHX, MTAD) or irrigation activation systems	Different irrigation protocols	Antimicrobial efficacy measured through (1) the total number of bacteria before and after irrigation and (2) positive result of bacterial samples after irrigation	Higher biofilm reduction and antimicrobial effects especially for activation methods	Some studies reported the disappearance of clinical symptoms	One included study showed success during endodontic treatment	Low (most of the studies showed high and uncertain risk of bias)

Chalub LO, 2023 [38]	To answer the question as to whether the use of ultrasonic irrigation (UI) results in better antimicrobial activity in root canal disinfection compared to conventional irrigation (CI)	Does ultrasonic irrigation result in better antimicrobial efficacy in root canal disinfection compared to conventional irrigation?”	Patients who have received conventional endodontic treatment	Ultrasonic irrigation	Syringe irrigation (conventional)	Antimicrobial effectiveness	Ultrasonic irrigation resulted in a better antimicrobial effect (statistically significant differences)	Not evaluated	Not evaluated	High (most of the studies with low risk of bias)
Meire MA, 2023 [39]	to critically appraise all available evidence regarding the efficacy of adjunct therapy for the treatment of AP, according to a population, intervention, comparison, outcome, time and study design (PICOTS)	In patients with AP in permanent teeth (P), what is the efficacy of any intracanal procedure going beyond chemomechanical preparation with instruments and traditionally delivered irrigants (I), in comparison with chemomechanical preparation with instruments and traditionally (syringe-needle based) delivered irrigants (C), in terms of tooth survival, radiographic and clinical (pain, tenderness, swelling and need for medication [analgesics, antibiotics])	General population, adult patients undergoing primary or secondary root canal treatment of a tooth with radiographic evidence of AP.	Adjunct therapy: any type of intracanal procedure going beyond chemomechanical preparation with instruments and traditionally delivered irrigants and carried out within the same visit. It includes irrigant activation methods/devices, light-mediated disinfection (photo-activated disinfection and direct laser irradiation) and the use of ozone.	Chemomechanical preparation with instruments and traditionally (syringe-needle based) delivered irrigants alone (excluding the use of intracanal medication).	Critical outcomes: tooth survival, pain, tenderness, swelling, need for medication (analgesics, antibiotics), radiographic evidence of reduction of apical lesion size (loose criteria), and radiographic evidence of normal periodontal ligament space (strict criteria). Secondary outcomes: tooth function (fracture, restoration longevity), need for further intervention, adverse effects (including exacerbation,	Not evaluated	No significant difference in the prevalence of pain 7-day post-treatment was demonstrated after meta-analysis on the two studies comparing diode laser irradiation with no adjunct therapy.	No statistically significant difference in healing after 12 months	Low (Principal reasons include serious risk of bias in the trials and imprecision due to the limited number of events and samples in the trials)

		healing of AP (O)'?				restoration integrity, allergy), oral health-related quality of life (OHRQoL) and presence of sinus tract.				
Quintana RM, 2023 [40]	To investigate whether antimicrobial photodynamic therapy (aPDT) after chemomechanical root canal disinfection (CD) yields a greater microbial load reduction than only CD.	Does aPDT used as a supplementary disinfection protocol in the primary endodontic treatment of teeth with chronic apical periodontitis provide a greater infection reduction than treatments without aPDT?	Participants with permanent teeth with a primary endodontic infection treated endodontically	Use of aPDT after CD	CD	Reduction of microbial load	Both disinfection methods resulted in significant colony formation units (CFU) reduction, although the overall effect was higher after aPDT (Z = 4.10; p < 0.001) than after CD alone (Z = 2.82; p = 0.005).	Not evaluated	Not evaluated	High (most of the studies with low risk of bias)
Weissheimer T, 2023 [41]	To compare the disinfectant ability of chlorhexidine (CHX) gel and sodium hypochlorite (NaOCl)	Is CHX gel as effective as NaOCl in disinfecting the root canal system?	Adult patients subjected to root canal treatment or retreatment presenting symptomatic or asymptomatic apical periodontitis	Root canal treatment using CHX gel as an adjunct substance during root canal preparation	Root canal preparation using liquid NaOCl	Primary: bacterial and/or endotoxin reduction; Secondary: periapical healing	There seems to be no difference in the disinfectant ability of CHX gel and NaOCl, but further research is necessary.	Not evaluated	Not evaluated	Low (most of the studies showed high and uncertain risk of bias)

Gobbo LB, 2024 [42]	To assess the impact of Passive Ultrasonic Irrigation on the periapical healing rate of primary root canal treatment compared to conventional syringe irrigation	Does Passive Ultrasonic Irrigation (PUI) during nonsurgical primary root canal treatment (P) significantly impact periapical healing (O) compared to conventional syringe irrigation or other activation techniques (C)?	Nonsurgical primary root canal treatment	Passive ultrasonic irrigation protocol (PUI)	Conventional syringe irrigation (CSI) and other irrigation techniques	Periapical healing	Not evaluated	The results showed that the use of PUI is more successful when compared to CSI on the periapical healing outcome	The findings related to the evaluated outcome consistently demonstrated that the use of PUI resulted in a higher percentage of periapical healing rate compared to CSI	Moderate (imprecision for some findings and considering the nature of risk of bias)
Hazrati P, 2024 [43]	To summarize studies assessing the effect of lasers on periapical lesion healing.	Could the use of lasers in RCT result in a greater decrease in the size of periapical lesions than conventional treatment methods?	Patients with a periapical lesion	Laser application in root canal therapy (RCT) as a sole or conjunctive treatment	Conventional endodontic treatment	Periapical lesion healing	Not evaluated	In all of the included studies, laser application outperformed the standard cleaning and shaping protocol; however, this improved or faster healing was not statistically significant in most studies.	Radiographic healing in conjunction with laser use, but without significant differences	Between moderate and low (imprecision for some findings and considering the nature of risk of bias)

*Abbreviations: RCT: Root Canal Therapy; aPDT: Antimicrobial Photodynamic Therapy; UAI: Ultrasonically Activated Irrigation; PUI: Passive Ultrasonic Irrigation; CSI: Conventional Syringe Irrigation; NaOCl: Sodium Hypochlorite; CHX: Chlorhexidine; EDTA: Ethylenediamide Tetraacetic Acid

Table S4. Detailed characteristics of the irrigants and the protocols used in endodontic therapy according to the reported findings [31–43].

Main characteristics of the irrigants and their protocols	Systematic Reviews/Metanalyses included													Number of times (the item is mentioned)
	Moreira RN, 2019 [31]	Neelakantan P, 2019 [32]	Silva EJNL, 2019 [33]	Anagnostaki E, 2020 [34]	Ruksakiet K, 2020 [35]	Ali NT, 2022 [36]	Tonini R, 2022 [37]	Chalub LO, 2023 [38]	Meire MA, 2023 [39]	Quintana RM, 2023 [40]	Weissheimer T, 2023 [41]	Gobbo LB, 2024 [42]	Hazrati P, 2024 [43]	
Retreatment	No	No	No	Yes	Yes	Yes	Yes	Yes	Yes	No	Yes	No	No	7
1 % NaOCl	Yes	Yes	No	No	Yes	No	Yes	Yes	No	Yes	Yes	No	No	7
Volume	NR	NR	N/A	N/A	10ml	N/A	10- 10.5ml	NR	N/A	NR	NR	N/A	N/A	2
2.5 NaOCl	Yes	Yes	Yes	Yes	Yes	Yes	Yes	Yes	Yes	Yes	Yes	Yes	Yes	13
Volume	NR	NR	8ml	NR	3- 15ml	5-8 ml	15- 25ml	NR	NR	NR	NR	NR	NR	4
3 % NaOCl 3%	No	No	No	No	No	Yes	No	No	Yes	Yes	No	Yes	Yes	5
Volume	N/A	N/A	N/A	N/A	N/A	5 ml	N/A	N/A	NR	NR	N/A	8ml	NR	3
5.25% NaOCl	No	No	Yes	No	Yes	Yes	No	Yes	Yes	Yes	No	Yes	No	7
Volume	N/A	N/A	6ml	N/A	2ml	6- 15ml	N/A	NR	NR	NR	N/A	6ml	N/A	4
6% NaOCl	Yes	No	No	No	No	Yes	No	Yes	No	No	Yes	No	No	4
Volume	NR	N/A	N/A	N/A	N/A	6-10ml	N/A	NR	N/A	N/A	NR	N/A	N/A	1
0.2% CHX	No	No	No	No	Yes	No	No	No	No	No	No	No	No	1
Volume	N/A	N/A	N/A	N/A	3ml	N/A	N/A	N/A	N/A	N/A	N/A	N/A	N/A	1
2% CHX	Yes	Yes	Yes	No	Yes	No	Yes	No	Yes	No	Yes	No	Yes	8
Volume	NR	NR	NR	NA	3- 15ml	N/A	1- 15ml	N/A	NR	N/A	NR	N/A	5ml	3
3% hydrogen peroxide	No	No	No	No	No	No	No	No	Yes	Yes	No	No	Yes	3
Volume	N/A	N/A	N/A	N/A	N/A	N/A	N/A	N/A	NR	10ml	N/A	N/A	NR	1
Saline solution	No	No	Yes	No	Yes	Yes	Yes	Yes	Yes	No	Yes	No	Yes	8
Volume	N/A	N/A	NR	N/A	3-15ml	45ml	1-15ml	NR	NR	N/A	NR	N/A	5ml	4
17% EDTA	Yes	No	Yes	Yes	No	Yes	No	No	Yes	Yes	Yes	Yes	Yes	9
Volume	NR	N/A	2-4ml	N/A	N/A	2-4ml	N/A	N/A	NR	NR	NR	2ml	1ml-5ml	4

Time (seconds)	NR	N/A	30-60	N/A	N/A	60	N/A	N/A	NR	NR	3 min	1 min	NR	4
Citric acid	No	No	No	No	No	No	No	No	No	No	No	No	No	0
Volume	N/A	N/A	N/A	N/A	N/A	N/A	N/A	N/A	N/A	N/A	N/A	N/A	N/A	0
MTAD	No	No	No	No	No	No	Yes	Yes	No	No	No	No	No	2
Volume	N/A	N/A	N/A	N/A	N/A	N/A	5ml	NR	N/A	N/A	N/A	N/A	N/A	5
HEDP	No	No	No	No	No	No	Yes	No	No	No	No	No	No	1
Volume	N/A	N/A	N/A	N/A	N/A	N/A	25ml	N/A	N/A	N/A	N/A	N/A	N/A	1
Activation	Yes	No	Yes	Yes	No	Yes	Yes	Yes	Yes	No	No	Yes	Yes	9
No activation	Yes	No	Yes	Yes	No	Yes	Yes	Yes	Yes	Yes	Yes	Yes	Yes	11
Time	NR	N/A	60	NA	NR	NR	60	NR	NR	NR	NR	NR	NR	1
Endoactivator	No	No	No	No	No	No	Yes	No	NO	No	No	No	No	1
Time	N/A	N/A	N/A	N/A	N/A	N/A	1 min	N/A	N/A	N/A	N/A	N/A	N/A	1
PUI	Yes	No	Yes	No	No	Yes	Yes	Yes	Yes	No	No	Yes	No	7
Time	NR	N/A	30-60	N/A	N/A	NR	180	NR	30s	N/A	N/A	30s-60s	N/A	4
F-File	No	No	No	No	No	No	Yes	No	No	No	No	No	No	1
Time	N/A	N/A	N/A	N/A	N/A	N/A	3 min	N/A	N/A	N/A	N/A	N/A	N/A	1
XP-Endofinisher	No	No	No	No	No	No	Yes	No	No	No	No	No	No	1
Time	N/A	N/A	N/A	N/A	N/A	N/A	3 min	N/A	N/A	N/A	N/A	N/A	N/A	1
Follow-up time	No	No	10- 19 months	6 months	1- 4 years	3 hrs-19 months	No	No	12 months	No	No	19 months	12 months	7
Radiographic analysis	N/A	N/A	Rx-CBCT	RX	N/A	RX-CBCT	No	N/A	Rx-CBCT	N/A	N/A	Rx-CBCT	Rx-CBCT	6
Irrigation time	NR	NR	NR	NR	NR	NR	NR	NR	NR	NR	NR	NR	NR	0
Shaping technique	Manual-Rotary	Rotary	Rotary-Reciprocating	Manual-Rotary-Reciprocating	Manual-Rotary	Rotary-Reciprocating	Manual-Rotary-Reciprocating	Manual-Rotary-Reciprocating	Manual-Rotary-Reciprocating	Manual-Rotary-Reciprocating	Manual-Rotary-Reciprocating	Rotary	Manual-Rotary-Reciprocating	13
Sample (n)	158	162	158	366	309	922	373	395	1146	320	214	501	420	13

Laser Type	N/A	N/A	N/A	Diodo, Er-YAG, Nd-YAG	N/A	N/A	N/A	N/A	Diodo,Nd-YAG-Er:YAG, Er,Cr:YSGG	Diodo,Nd-YAG-Er:YAG, LED	N/A	N/A	Diodo-Eh,Cr:YSGG -Nd-YAG	4
Photosensitizer concentration (mg/mL)	N/A	N/A	N/A	0,025-10	N/A	N/A	N/A	N/A	NR	No	N/A	N/A	No	1
Incubation time (min)	N/A	N/A	N/A	2--5	N/A	N/A	N/A	N/A	NR	No	N/A	N/A	No	1
Power (mW)	N/A	N/A	N/A	40-1000	N/A	N/A	N/A	N/A	100-1250	40-8400	N/A	N/A	40-2000	4
Irradiation time (sec)	N/A	N/A	N/A	30-300	N/A	N/A	N/A	N/A	20-90	20-240	N/A	N/A	60-240	4
Tip (µm)	N/A	N/A	N/A	200-750	N/A	N/A	N/A	N/A	200-600	200-500	N/A	N/A	200-320	4
Number of sessions	N/A	N/A	N/A	1	N/A	N/A	N/A	N/A	NR	No	N/A	N/A	No	1

Abbreviations: NA: Not applicable; NR: Not reported

Table S5. Primary study overlap in the included SR-MAs (n=74) [21,31–43, 45–117]

Primary studies included	Systematic Reviews/Metanalyses included													Number of times included
	**Moreira RN, 2019 [31]**	**Neelakantan P, 2019 [32]**	**Silva EJNL, 2019 [33]**	**Anagnostaki E, 2020 [34]**	**Ruksakiet K, 2020 [35]**	**Ali NT, 2022 [36]**	**Tonini R, 2022 [37]**	**Chalub LO, 2023 [38]**	**Meire MA, 2023 [39]**	**Quintana RM, 2023 [40]**	**Weissheimer T, 2023 [41]**	**Gobbo LB, 2024 [42]**	**Hazrati P, 2024 [43]**	
Burleson A, 2007 [68]	X													1
Carver K, 2007 [52]	X							X						2
Beus C, 2012 [53]	X							X						2
Paiva SS, 2012 [69]	X													1
Paiva SS, 2013 [70]	X													1
Gomes BP, 2009 [54]		X									X			2
Xavier AC, 2013 [46]		X			X						X			3
Marinho AC, 2014 [55]		X									X			2
Marinho AC, 2015 [56]		X									X			2
Liang YH, 2013 [45]			X			X			X			X		4
Herrera DR, 2017 [71]			X											1
Nakamura VC, 2018 [47]			X			X		X						3
Garcez AS, 2010 [72]				X										1
Jurič IB, 2014 [73]				X										1
Yoo YJ, 2014 [74]				X										1
Arslan H, 2017 [75]				X										1
Asnaashari M, 2017 [76]				X										1
Pourhajibagher M, 2017 [77]				X										1
de Miranda RG, 2018 [78]				X										1
Doğanay Yıldız E, 2018 [79]				X										1
Nabi S, 2018 [80]				X										1
Arslan H, 2019 [81]				X										1
Morsy DA, 2018 [48]				X					X	X				3
Coelho MS, 2019 [82]				X										1
Dagher J, 2019 [83]				X										1
Doğanay Yıldız E, 2019 [84]				X										1
Genc Sen O, 2019 [85]				X										1

Lopes LPB, 2019 [86]	X									1
Nunes EC, 2020 [87]	X									1
Ringel AM, 1982 [88]		X								1
Kuruvilla JR, 1998 [89]		X								1
Ercan E, 2004 [90]		X								1
Vianna ME, 2006 [57]		X					X			2
Rôças IN, 2016 [67]		X			X					2
Zandi H, 2016 [58]		X		X						2
Zandi H, 2019 [91]		X								1
Johnson KL, 2011 [59]			X		X					2
Tang Z, 2015 [49]			X			X		X		3
Jain A, 2017 [92]			X							1
Middha M, 2017 [60]			X			X				2
Topçuoğlu HS, 2018 [93]			X							1
Orozco EIF, 2019 [50]			X	X	X					3
Aveiro E, 2020 [61]			X		X					2
Ballal NV, 2020 [51]			X	X	X					3
Malkhassian G, 2009 [94]				X						1
Huffaker SK, 2010 [21]				X						1
Ballal NV, 2019 [95]				X						1
Bellingham A, 2011 [96]					X					1
Lu CH, 2017 [97]					X					1
Bilgin B, 2020 [98]					X					1
Palanisamy R, 2020 [99]					X					1
Shehab Aldean AA, 2020 [100]					X					1
Barciela B, 2019 [101]						X				1
Guimarães LDS, 2021 [102]						X				1
Kaplan T, 2021 [103]						X				1
Kist S, 2017 [104]						X				1
Koba K, 1999 [62]						X			X	2
Masilionyte M, 2018 [63]						X			X	2
Martins MR, 2014 [64]						X			X	2
Souza MA, 2021 [105]						X				1

Verma A, 2020 [65]	X			X		2
Pietrzycka K, 2011 [106]	X					1
Garcez AS, 2008 [107]		X				1
Garcez AS, 2008 (2) [66]		X			X	2
Granevik Lindström M, 2017 [108]		X				1
Rabello DGD, 2017 [109]		X				1
Razumova SN, 2019 [110]		X				1
Zorita-García M, 2019 [111]		X				1
Di Taranto V, 2022 [112]		X				1
Barbosa-Ribeiro M, 2016 [113]			X			1
Dalaei Moghadam M, 2021 [114]					X	1
Shaheed AA, 2020 [115]					X	1
Martins MR, 2013 (2) [116]					X	1
Karakov KG, 2018 [117]					X	1
